# Effect of Florpyrauxifen-Benzyl on Methane-Metabolizing Microbial Community in Rice Rhizosphere Soil

**DOI:** 10.3390/microorganisms14061228

**Published:** 2026-05-29

**Authors:** Mingrui Yuan, Fengshan Yang, Pan Wang, Haiyan Fu, Zhijian Ge, Zongyang Zhang, Chunguang Liu

**Affiliations:** Engineering Research Center of Agricultural Microbiology Technology, Ministry of Education & Heilongjiang Provincial Key Laboratory of Ecological Restoration and Resource Utilization for Cold Region & Key Laboratory of Microbiology, College of Heilongjiang Province & School of Life Sciences, Heilongjiang University, Harbin 150080, China; yuanmingrui0321@163.com (M.Y.); yangfengshan@hlju.edu.cn (F.Y.); wpan222@163.com (P.W.); fuhaiyan@hlju.edu.cn (H.F.); ge20000113@163.com (Z.G.); 2241764@s.hlju.edu.cn (Z.Z.)

**Keywords:** rice rhizosphere soil, methane emissions, methanotrophs, methanogens, florpyrauxifen-benzyl

## Abstract

Florpyrauxifen-benzyl is a newly developed aryl pyridine carboxylic acid ester herbicide for rice paddies, and its effects on microbial communities remain largely unexplored. Rice paddy fields represent the largest source of anthropogenic methane emissions. However, whether the overapplication of florpyrauxifen-benzyl causes changes in methane-metabolizing microorganisms and consequently elevates methane emissions has not been thoroughly investigated. In this study, we investigated the effects of applying florpyrauxifen-benzyl at increasing concentrations on methane-metabolizing microorganisms, using high-throughput sequencing of functional genes involved in methane metabolism. The results were validated via quantitative fluorescence PCR. The application of florpyrauxifen-benzyl at five times the recommended dose significantly increased methane emissions. Co-occurrence network analysis revealed a more complex network structure in the methanogenic community. Furthermore, random forest modeling and LEfSe analysis indicated that *Methylocystis*, *Methylosinus*, *Methanolobus*, *Methanosphaera*, and *Methanococcoides* play key roles in maintaining the stability of methane-metabolizing microbial communities.

## 1. Introduction

Chemigation plays a significant role in the rapid loss of biodiversity, which is a major global threat to the functioning and services of natural and agro-ecosystems [1]. Herbicides, being important external chemicals, serve as a convenient, effective, and powerful tool for disrupting or eliminating weed growth [2]. However, only 20–30% of the herbicide’s active ingredient is applied at the intended location, while the remaining 60–70% is transferred from the site of action to the surrounding environment (soil, water, and air) [3]. This results in reduced biological activity and poses a significant risk to ecosystems [4]. For example, mesotrione is a selective triketone herbicide known to have negative effects on soil microorganisms, especially at high doses [5]. The impact of different herbicides on soil microbial communities and their functions varies depending on factors such as chemical structure, concentration, and toxicity. Additionally, the effects of herbicides on microorganisms can differ under different soil environmental conditions [6]. Herbicides significantly alter the composition of soil microbial communities and their carbon utilization patterns, which in turn have profound implications for soil fertility and climate [7]. Given the widespread use of herbicides in modern agricultural practices, researchers have started investigating the potential impact of these chemicals on the soil carbon cycle [8]. Florpyrauxifen-benzyl is a herbicide developed by Corteva in recent years. It belongs to the aryl pyridine carboxylate class of herbicides. Unlike other hormonal herbicides, florpyrauxifen-benzyl has a unique mechanism of action [7,8,9]. However, as an emerging herbicide, current research has primarily focused on its weed control efficacy, safety to current and succeeding crops, soybean sensitivity, soil adsorption and transport, and the responses of aquatic plants. Yet, its effects on the methane-metabolizing microbial community, the expression of functional genes involved in methane metabolism, methane emissions, and soil physicochemical properties in the rhizosphere soil of paddy fields remain unclear. Therefore, further studies on the soil methane-metabolizing microbial community are needed to elucidate its influence on sustainable soil utilization and its potential impacts on microorganisms involved in soil carbon cycling.

The northeast black soil region stands out as the most important commercial grain base in China [10]. The region experiences an average annual temperature ranging from 0.5 °C to 6 °C, characterized by severe and prolonged winters. The soil freezes deeply, with a freezing time of about 120 to 200 days (https://www.cas.cn/zt/kjzt/htlc/jstx/202105/t20210512_4787756.shtml (accessed on 13 March 2021)). Rice, being one of the world’s most important staple food crops, is expected to experience a 24 percent increase in global demand over the next 20 years [11]. China, being a major rice-growing country, contributes to 28.1% of the world’s rice production [12]. In Heilongjiang, it is known for its black soil. As the top grain-producing province in China, it maintains an annual planting area of grain crops exceeding 220 million mu and has consistently produced over 150 billion jin of grain for eight consecutive years, ranking first nationwide for sixteen years. (https://nynct.hlj.gov.cn/nynct/c115443/public_tt_time.shtml (accessed on 11 February 2026). It is worth noting that rice fields are a significant source of anthropogenic methane emissions, and the release of methane poses a serious threat to the global climate [13]. Rice fields are responsible for emitting 25–300 Tg of methane annually, making up 7–17% of the total global methane emissions. As a result, it is crucial to actively seek methods to reduce soil methane emissions from rice fields [14]. The amount of methane released from these fields is determined by the equilibrium between methane production and methane oxidation, which is influenced by various factors. These factors include both biological elements such as methanogens and methanotrophs, as well as non-biological factors like soil water-holding capacity and temperature. The interactions between these factors also play a significant role [15].

Methanogens and methanotrophs play a crucial role in CH_4_ metabolism in paddy soils. The main enzyme involved in methanogenesis is methyl coenzyme M reductase (MCR), which is known for its conservatism. So far, there has been no evidence of horizontal gene transfer in MCR. Therefore, the functional gene *mcrA* can be utilized to assess the diversity of methanogens in specific environments [16]. Methane monooxygenase is the key enzyme responsible for methane oxidation. Currently, the *pmoA* gene, which encodes the *β* subunit of the methane monooxygenase protein, is widely used to detect methanotrophs in environmental samples [17,18]. Research on the functional genetics of methanotrophs has primarily focused on various environments such as rice paddies, peatlands, wetlands, landfills, and areas rich in oil and coal [19]. There are three main pathways for CH_4_ production in paddy soils: the hydrogenotrophic, acetoclastic, and methylotrophic pathways [20]. CH_4_ is oxidized by methane monooxygenase to form methanol, which is then further oxidized by methanol dehydrogenase (MDH) in the periplasmic space to form formaldehyde. Eventually, formaldehyde is converted into CO_2_ and H_2_O through the RuMP, CBB, and serine cycles, respectively [21]. The emissions of CH_4_ will depend on the balance between methane oxidation and methane production.

Currently, most studies have focused on investigating the mechanisms of adding different carbon sources or electron acceptors in response to CH_4_ emissions [22,23]. While herbicides are widely used in agricultural practices, their impact on CH_4_ emissions from paddy soils has received limited attention. The application of herbicides is equivalent to adding a carbon source to the soil. During the degradation of herbicides, methanol and CO_2_ are produced [24,25]. The methanogenic grows with methanol and CO_2_ to form CH_4_. This process is linked to the methane metabolism pathway. In this study, florpyrauxifen-benzyl is applied to explore its effects on CH_4_ emissions under various concentrations. We measured the dynamics of CH_4_ emissions and analyzed the community composition of methane-metabolizing microorganisms using high-throughput sequencing of carbon cycle functional genes. Our hypotheses were as follows: (1) The application of florpyrauxifen-benzyl ester can affect methane emissions in paddy soil. (2) Florpyrauxifen-benzyl would disrupt the structure of methane-metabolizing microbial communities. (3) Florpyrauxifen-benzyl would have contrasting effects on the diversity of methanotrophs and methanogens. The findings from this experiment will contribute to a better understanding of the impact of herbicides on the carbon cycle and offer insights for mitigating methane emissions from paddy soil.

## 2. Materials and Methods

### 2.1. Test Soil Samples

Soil samples were obtained from the experimental rice field (126.6° E, 45.9° N) at the Hulan Campus of Heilongjiang University. The inter-root soil of rice, which had not been treated with chemical herbicides for 2 years, was collected. Fresh soil was collected using the five-point sampling method. The soil was dried at 36 °C until constant weight, and then ground and passed through 40-mesh and 60-mesh sieves, and the sieved soil was stored for subsequent use. The soil collected in this experiment was chernozem (black calcareous soil). The samples contained 8.31 g/kg total nitrogen (TN), 320.62 mg/kg total phosphorus (TP), 15.50 g/kg total potassium (TK), 153.99 g/kg organic matter (OM), and had a water content (MC) of 1.92% with a pH of 6.00. The soil density was 2.5 g/cm^3^ and the soil bulk density was 1.2. The mean annual temperature in the area is 3.6 °C, with a mean annual rainfall of 569.1 mm. This region is located in the second thermocline. Fresh soil was collected using the five-point sampling method. The soil was dried at 36 °C until its weight stabilized, ground, sieved through a 40-mesh and 60-mesh sieve, and then collected.

### 2.2. Experimental Design

Pot experiment design: The rice variety used was Wuyoudao 4. The pot dimensions were 14.5 cm × 15.5 cm × 33 cm, with each pot containing 4 kg of paddy soil. The calibration procedure for the detector was as follows: first, turn on the device outdoors and let it stand for 10 min to ensure normal operation. After startup, use the built-in calibration option to perform methane calibration in the outdoor air. The herbicide used in this experiment was florpyrauxifen-benzyl, a foliar herbicide, applied during the seedling stage when rice was at the two-leaf, one-heart stage. The study period lasted 30 days after herbicide application, during which the rice plants were at the tillering stage.

Four different concentrations of florpyrauxifen-benzyl were applied to the potted rice. The analytical standard, florpyrauxifen-benzyl (3% emulsion), was obtained from Corteva (Johnston, IA, USA). Depending on crop type, weed density, and grass age, the globally recommended effective application of florpyrauxifen-benzyl ranges from 5 to 50 g a.i. hm^−2^. Three florpyrauxifen-benzyl treatment doses, F1: 5 g a.i. hm^−2^, F5: 25 g a.i. hm^−2^, and F10: 50 g a.i. hm^−2^, were used in the experiment. The control (F0) was not treated with florpyrauxifen-benzyl.

During the experiment, soil samples were collected and processed on days 1, 5, 15, and 30. Rice seeds (Wuyoudao 4, China) were grown in nursery pots for 30 days until they reached the three-leaf, one-heart stage. They were then moved into fixtures maintained at a constant hothouse incubation temperature of 25 °C. The fixtures were sealed after transplanting the rice seedlings. Four florpyrauxifen-benzyl concentration gradients and four-time gradients were used in the experiment. Three replicates were performed for each treatment. This study conducted a total of 48 potted rice seedling experiments. Each pot was sealed with a plastic bucket lid. A soil sampler was used to collect 10–20 cm of inter-root rice soil. Each sampling step was destructive. Methane gas was collected from the device on days 1, 5, 15, and 30 from 9 to 11 am [26]. The gas samples were analyzed using a methane detector (Zhongantan Co., Ltd., Zhengzhou, China). Cumulative and net CH_4_ emission fluxes were calculated and analyzed.

### 2.3. Determination of Florpyrauxifen-Benzyl in Soil Samples

Soil samples were sieved through 40- and 60-mesh sieves, and 5 g of soil and 2 g of diatomaceous earth(Baisi Chemistry, Hangzhou, China) were added to a 50 mL centrifuge tube and mixed well. Next, 10 mL of a hexane(Kemiou, Zhuhai, China):acetone(Kemiou, China) (1:1, *v*/*v*) solvent mixture was added, oscillated, extracted via ultrasonic extraction for 5 min, and centrifuged at 5000 r/min for 5 min. Then, 10 mL of the supernatant was pipetted into a culture tube and blow-dried under nitrogen. The remaining supernatant was placed in a culture tube and blow-dried again; this process was repeated twice. Methanol (Thermo Fisher, Waltham, MA, USA) (2 mL) was fixed, dissolved via ultrasonication, passed through a 0.22 μm filter membrane, and the obtained samples were placed in 2 mL centrifuge tubes. The liquid in the centrifuge tubes was filtered using SPE columns to remove pigments and other impurities, and stored at −20 °C until further analysis [27].

The florpyrauxifen-benzyl residue was detected by ultra-performance liquid chromatography (UPLC) [28]. The samples were passed through an ultra-performance liquid chromatograph with optimal chromatographic conditions: a Waters Acquity UPLC BEH C18 column (1.7 μm, 2.1 × 50 mm), a detector wavelength of 230 nm, a column temperature of 40 °C, a pump flow rate of 0.3 mL/min, a mobile phase of acetonitrile (Thermo Fisher, USA):water (3:1), an injection volume of 3 μL, and a retention time of 2.916 ± 0.05 min. A standard curve was plotted according to the relationship between the measured peak area and the concentration of the standards, and a regression equation was established. The spiked recovery of florpyrauxifen-benzyl was 97.5 ± 8.9%.

### 2.4. Tests on Soil Physical and Chemical Characteristics

Experiments were conducted to determine soil TN, TP, TK, OM, MC, and pH. The experiments were carried out using the Kjeldahl nitrogen determination method for soil TN, the molybdenum antimony sulphate antimony colorimetric method for soil TP, the flame photometer method for soil TK alkalinity, the volumetric method with potassium dichromate–external heating for soil OM, the drying method to detect MC, and a pH meter for soil pH [29].

### 2.5. High-Throughput Sequencing of CH_4_ Metabolism Functional Genes

High-throughput sequencing was performed of functional genes involved in CH_4_ metabolism. Total genomic DNA was extracted from the rice soil (0.5 g) using the TGuide S96 magnetic bead method with a soil genomic DNA extraction kit. The concentration of extracted DNA was measured using an enzyme marker. The integrity was tested via electrophoresis using agarose at a concentration of 1.8%. After passing quality control, polymerase chain reaction amplification of *pmoA* and *mcrA* was carried out using primers F:5′-TGGGGYTGGACCTAYTTCC-3′, R:5′-CCGGCRCRACGTCCTTACC-3′, and F:5′-GGTGGTGTMGGATTCACACARTAYGCWACAGC-3′, R:5′-TTCATTGCRTAGTTWGGRTAGTT-3′, respectively, on an EDC-810 PCR instrument (Eastwin Life Sciences, Inc., Suzhou, China). The cycling program consisted of a pre-denaturation step at 94 °C for 5 min. This was followed by 35 cycles of denaturation at 94 °C for 1 min, annealing at 56 °C for 1 min, and extension at 72 °C for 1 min. After the cycles, a final extension was performed at 72 °C for 10 min, followed by a hold at 4 °C. The PCR amplification products were then purified, quantified, and homogenized to create sequencing libraries. These libraries underwent quality control before being sequenced using the Illumina Novaseq platform (Biomarker Technologies, Beijing, China) [28,29,30,31].

### 2.6. Quantitative Fluorescence PCR for CH_4_ Metabolism Functional Genes

The assay was performed using a CFX96 real-time fluorescence quantitative PCR instrument (Bio-Rad, Hercules, CA, USA) and the fluorescent dye SYBR Green (Takara, Kyoto, Japan) for qPCR [32]. The test was performed for absolute quantification of the CH_4_ cycle functional genes *pmoA* and *mcrA* [28]. The reaction volume for the amplification process contained 7.5 μL of 2×SYBR Green Mix, 1 μL of gDNA, 0.7 μL each of forward and reverse primer reaction systems, and 15 μL ddH_2_O. The amplification efficiencies of the target genes *pmoA* and *mcrA* were 91.96% and 102.09%, respectively, with R^2^ values of 0.999. Melting curve analysis confirmed the specificity of the target gene amplification, and the results showed single peaks. The parameters were as follows: *pmoA* annealing Tm: 36 °C; *pmoA* melting Tm: 87 °C; *mcrA* annealing Tm: 34 °C; *mcrA* melting Tm: 86 °C; *pmoA* plasmid size: 3018 bp; *mcrA* plasmid size: 3170 bp.

### 2.7. Statistical Analysis

Equation (1) was used to describe the dissipation kinetics of florpyrauxifen-benzyl, Equation (2) was used to calculate the half-life, and the daily methane flux was calculated as shown in Equation (3) [31].

(1) $$C\left(t\right)=C_{0}e^{-kt}$$

(2) $$T_{1/2}=(ln2)/k$$

(3) F=ρ×V/m×dc/dt×273/(273+T)×12/16

*C*(*t*) is the herbicide residue in the soil at time *t*; and *C*_0_ is the herbicide residue in the soil at the initial time.

*k* is calculated using Equation (1) and then substituted into Equation (2) to obtain *T*_1/2_.

*F* (mg C kg^−1^day^−1^) is the CH_4_ flux; *ρ* is the density of CH_4_ (0.717 kg m^−3^) at standard temperature and pressure (STP); *V* (m^3^) is the headspace volume; *m* (kg) is the dry soil weight; dc/dt (ppm d^−1^) is the change in CH_4_ concentration per unit time (d); and *T* is the incubation temperature.

Sequencing experiments were conducted on the Illumina NovaSeq 6000 platform (Illumina, Inc., San Diego, CA, USA). Raw Reads obtained from sequencing were filtered using Trimmomatic v0.33 software to obtain Clean Reads without primer sequences. Clean Reads from each sample were spliced using Usearch v10 software, and then the spliced data were length filtered based on the different regions’ length range. Chimeric sequences were identified and removed using UCHIME v4.2 software to obtain the final Effective Reads. Sample volumes were appropriately diluted and sequenced for data analysis. The software QIIME (version 2.0) was used to assess the alpha diversity index of the soil samples’ microbial community. Differences in the alpha diversity index between treatments were evaluated using a *t*-test. Beta diversity analyses were conducted using the QIIME (version 2.0) software to analyze changes in species composition at temporal and spatial scales. The binary Jaccard algorithm was primarily used for beta diversity analysis to calculate the distance between samples and obtain the beta value. Between-group differences were analyzed by plotting a sample principal coordinate analysis (PCoA) using the R (version 4.5.3) language platform. The random forest package (version 4.7-1.1) in R was used to predict the random forest model for methane metabolizing microorganisms and assess the significance of the variable factors. The Gephi software (version 0.9.2) was used to conduct a Spearman’s rank correlation analysis based on changes in OTU, and a correlation network was constructed by screening data with a correlation greater than 0.6 and a *p*-value less than 0.05. The collapse_table (version 6.1.0). py script in Python (version 3.14.2) was used to make FAPROTAX functional predictions at the microbial genus level and determine more detailed microbial community functions. Redundancy analysis of environmental factors and microbial matrices, as well as variance decomposition analysis, were performed using the Vegan package (version 2.6-8) in R. The linkET package (version 0.0.7.4) in R was used to conduct Mantel’s test and correlation analysis on the data matrix and factor matrices to demonstrate the correlation between the microbial matrix and the environmental factor matrix. Structural equation modeling was performed using the AmosGraphicsCLI software (version 24.0). All statistical analyses were conducted using SPSS software (version 26.0) (IBM, New York, NY, USA). Statistical significance was defined as *p* < 0.05 for differences, *p* < 0.01 for significance, and *p* < 0.001 for extreme significance.

## 3. Results

### 3.1. Residue and Degradation Dynamics of Florpyrauxifen-Benzyl in Rice Inter-Root Soils

The residues and degradation of clopyralid in rice rhizosphere soil are as follows. The half-life of F1, F5, and F10 in the soil was measured to be 23.90 d, 19.80 d, and 21.00 d, respectively. After 30 days of incubation, the degradation rates of F1, F5, and F10 reached 62.0%, 67.6%, and 62.8%, respectively. The residual dynamics of florpyrauxifen-benzyl in the soil followed a negative exponential function (Table 1).

### 3.2. Effect of Florpyrauxifen-Benzyl on CH_4_ Emissions

**Figure 1:**
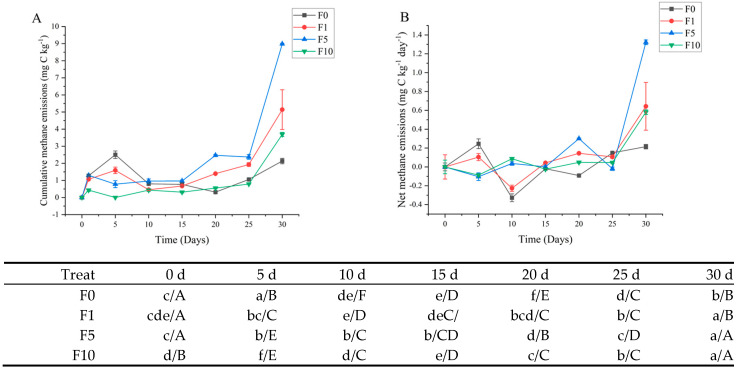
Effect of florpyrauxifen-benzyl on CH_4_ emissions. (**A**) cumulative CH_4_ emissions. (**B**) net CH_4_ emissions. F1 is 0.1 mg/L, F5 is 0.5 mg/L, and F10 is 1.0 mg/L (AVE ± SD, *n* = 3). Note: Lowercase letters indicate significance for (**A**), and uppercase letters indicate significance for (**B**); *p* < 0.05.

The effects of different concentrations of flupyradifurone-benzyl on methane emissions are as follows (Figure 1). Cumulative CH_4_ emissions were in the order of F0 > F1 > F5 > F10 on D0–5 of the experiment. At D5–10, the cumulative CH_4_ emissions were initially in the order of F0 > F1 > F5 > F10; however, on D7, the cumulative CH_4_ emissions from F5 gradually increased and exceeded those from F1 and F0. Cumulative CH_4_ emissions varied steadily from D10–15, with cumulative CH_4_ emissions under treatment F5 being greater than those under treatments F0, F1, and F10. From D15–20, cumulative CH_4_ emissions from the treatment groups showed an upward trend, whereas those from the control group showed a downward trend. At D20–25, the CH_4_ emissions of F5 were higher than those of F1, F10, and F0, with similar cumulative CH_4_ emissions in F10 and F0. At D25–30, the cumulative CH_4_ emissions showed an upward trend in the order of F5 > F1 > F10 > F0. The maximum cumulative CH_4_ emission reached 8.98 mg C kg^−1^ (Figure 1A).

The net CH_4_ emissions followed a trend similar to that of the cumulative CH_4_ emissions. At D0–D5, an increasing trend was observed in F0 and F1, and a decreasing trend in F5 and F10. At D5–D10, the trend in net CH_4_ emissions was contrary to that at D0–D5; at D10–D15, F5 and F10 showed a small decreasing trend, whereas F0 and F1 showed a large increasing trend. On D15, the net CH_4_ emissions of the treatment and control groups were similar. At D15–D20, net CH_4_ emissions increased in the treatment group but decreased in the control group; at D20–D25, net CH_4_ emissions of F1 and F10 were essentially unchanged, whereas they decreased under the F5 treatment and increased under the F0 treatment. On days 25–30 of the experiment, a substantial upward trend was observed in all treatment groups and a slow increase in the control group, with the net CH_4_ emissions in the order F5 > F1 > F10 > F0. Net CH_4_ emissions reached 1.32 mg C kg^−1^day^−1^ within 30 d of the experiment (Figure 1B). Methane emissions were higher in the treatment group than in the control group on D30 of the trial than on D1. In summary, florpyrauxifen-benzyl leads to increased methane emissions from rice inter-root soils. Application of florpyrauxifen-benzyl at 5 times the recommended dose resulted in significantly higher methane emissions than the other treatment groups (Figure 1).

### 3.3. Effect of Florpyrauxifen-Benzyl on the Structure of Microbial Communities Related to CH_4_ Metabolism

**Figure 2:**
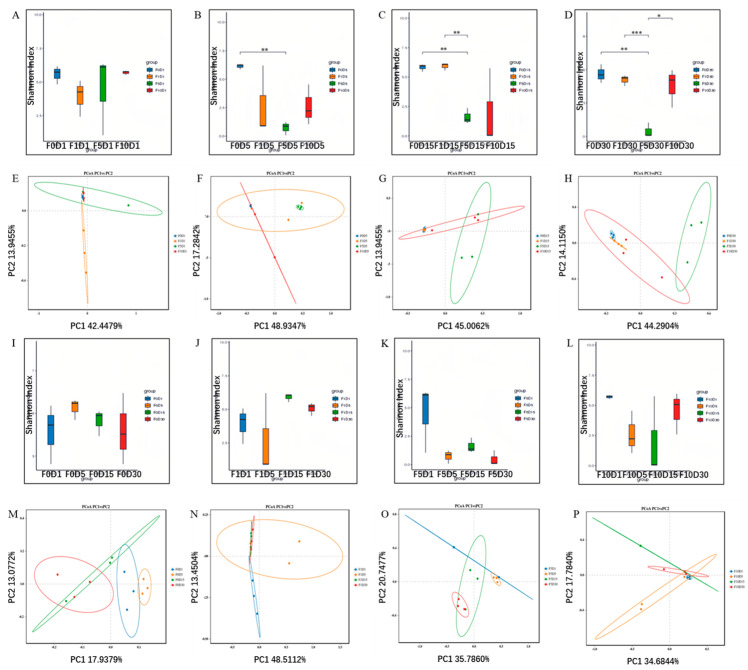
Effect of increasing concentrations of florpyrauxifen-benzyl on the diversity of methanotrophs. (**A**–**D**): effects of increasing concentrations of florpyrauxifen-benzyl on the alpha diversity of methanotrophs. (**E**–**H**): effects of increasing concentrations of florpyrauxifen-benzyl on the beta diversity of methanotrophs. (**I**–**L**): effect of variation in days on the alpha diversity of methanotrophs. (**M**–**P**): effect of variation in days on the beta diversity of methanotrophs. (*** *p* < 0.001, ** 0.001 < *p* < 0.01 and * 0.01 < *p* < 0.05).

The effects of florpyrauxifen-benzyl concentrations on methane-metabolizing microorganisms are as follows. (Figure 2). A total of 3,838,068 and 3,885,603 methanotroph and methanogen sequences, respectively, were obtained. The reads were clustered at a 97% similarity level to obtain Operational Taxonomic Units (OTUs). Methanotrophs and methanogens showed 364 and 466 OTUs, respectively (Figure S1). By counting the number of sample sequences at each stage of data processing and assessing the quality of the data, rank abundance curves indicated a high degree of species homogeneity, allowing for subsequent data analyses (Figure S2). The effect of florpyrauxifen-benzyl on the alpha and beta diversity of methane-metabolizing microorganisms was experimentally investigated (Figure 2). The alpha diversity of the methanotroph colony was found to be significantly higher at D15 compared to F0 for F1 and F5. Additionally, at D30, the alpha diversity of F10 was significantly higher compared to F5. Furthermore, the alpha diversity was significantly higher at D15 compared to D5 under F1 treatment. On the other hand, the alpha diversity of D30 was significantly reduced under F5 treatment compared to D1, D5, and D15 (Figure 2). The experimental principal coordinates analysis of methanotrophs flora for different treatments showed that D15 and D30 exhibited changes with florpyrauxifen-benzyl application dose in three groups of replicated trials. The degree of dispersion between the different florpyrauxifen-benzyl treatment groups was large, and all florpyrauxifen-benzyl treatment groups were significantly different from each other (Figure 2). Moreover, within the same florpyrauxifen-benzyl dose treatment, there was a significant difference between groups for D30 compared to D1 under F5 treatment. Similarly, under F10 treatment, the difference between groups was significant for D15 compared to D1. The alpha diversity was found to be significantly greater in F10 than in F5, except at D30, where it decreased under the other treatments (Figure S3). Alpha diversity did not change significantly under different temporal treatments. Experimental principal coordinate analysis of the methanogen flora for different treatments showed the following. At D30, the difference between groups was significant for F5 compared to F0 and F1. Under F5 treatment, D30 differed significantly compared to D1 and D5. In summary, florpyrauxifen-benzyl affects the alpha and beta diversity of methane-metabolizing microorganisms, with florpyrauxifen-benzyl treatment having a greater impact on the diversity of the methanotroph flora. Compared to F0, F5 and F10 had a greater effect on methane-metabolizing microorganisms. The change in diversity of the methanotroph flora with time was significant under F1 and F5 treatments. The change in the methanogen flora with time was not significant. Trends in alpha and beta diversity of methanogens and methanotrophs under florpyrauxifen-benzyl treatment are contrary (Figure 2).

At the phylum level, the composition of methanotrophs differed significantly from that of methanogens, with the vast majority of methanotrophs in the inter-root soils of the flooded rice fields belonging to Proteobacteria and a very small proportion belonging to candidate-division-NC10 (Figure S4A). In contrast, all the methanogens in the inter-root soil of the flooded rice fields belonged to Euryarchaeota (Figure S4B). The dominant genera (relative abundance > 1%) among methanotrophs were *Methylocystis* (60.48%) and *Methylococcus* (8.33%) (Figure S3C). The dominant genera (relative abundance > 1%) among methanogens were *Methanoregula* (23.65%), *Methanobacterium* (17.23%), *Methanolobus* (9.90%), *Methanococcoides* (7.38%), *Methanomassiliicoccus* (3.66%), *Methanosaeta* (1.38%), *Methanosarcina* (1.34%), and *Methanospirillum* (1.30%) (Figure S4D).

The effects of florpyrauxifen-benzyl on the species composition of methane-metabolizing microorganisms are as follows (Figure S5). For methanotrophs, a total of five types of methanotrophs were found under herbicide treatment, namely *Methylocystis*, *Methylococcus*, *Methylosinus*, *Methylobacter*, and *Methylocapsa*. The abundance of D15 *Methylococcus* increased significantly under F1 treatment. Among them, the abundance of D30 *Methylosinus* increased significantly under F5 treatment. At D15, the number of *Methylococcus* increased significantly, and the abundance of *Methylocystis* decreased significantly. At D30, the abundance of *Methylocystis* decreased significantly. *Methylocapsa* and *Methylobacter* showed a significant increasing trend in abundance in the F1D5 and F5D30 treatment groups, respectively (Figure S5A).

For methanogens, a significant decrease in the abundance of *Methanococcoides* and a significant increase in the abundance of *Methanoculleus*, *Methanolinea*, *Methanogula*, and *Methanosphaera* were observed under F1 treatment. Under F5 treatment, the abundance of *Methanoculleus*, *Methanolinea*, and *Methanomassiliicoccus* decreased significantly, and the abundance of *Methanococcoides* and *Methanolobus* increased significantly. The abundance of *Methanococcoides* and *Methanobacterium* significantly increased under F10 treatment. The number of *Methanococcoides*, *Methanomicrobiaceae_strain_EBac* increased significantly on day D1. At D5, there was a significant decrease in the abundance of *Methanocella*, *Methanoculleus*, *Methanomicrobiaceae_strain_EBac*, and *Methanosphaerula*. At D5, the abundance of *Methanolobus* increased significantly. At D15, there was a significant decrease in the abundance of *Methanoculleus*, *Methanomassiliicoccus*, and *Methanosphaerula*. There was also a significant increase in the abundance of *Methanococcoides*, *Methanogula*, *Methanosphaera*, and *Methanospirillum*. At D30, the abundance of *Methanobacterium* decreased significantly and the abundance of *Methanolobus* increased significantly (Figure S5B). The phylogenetic trees of methanogenic and methanotrophic bacteria are presented (Figure S6).

**Figure 3:**
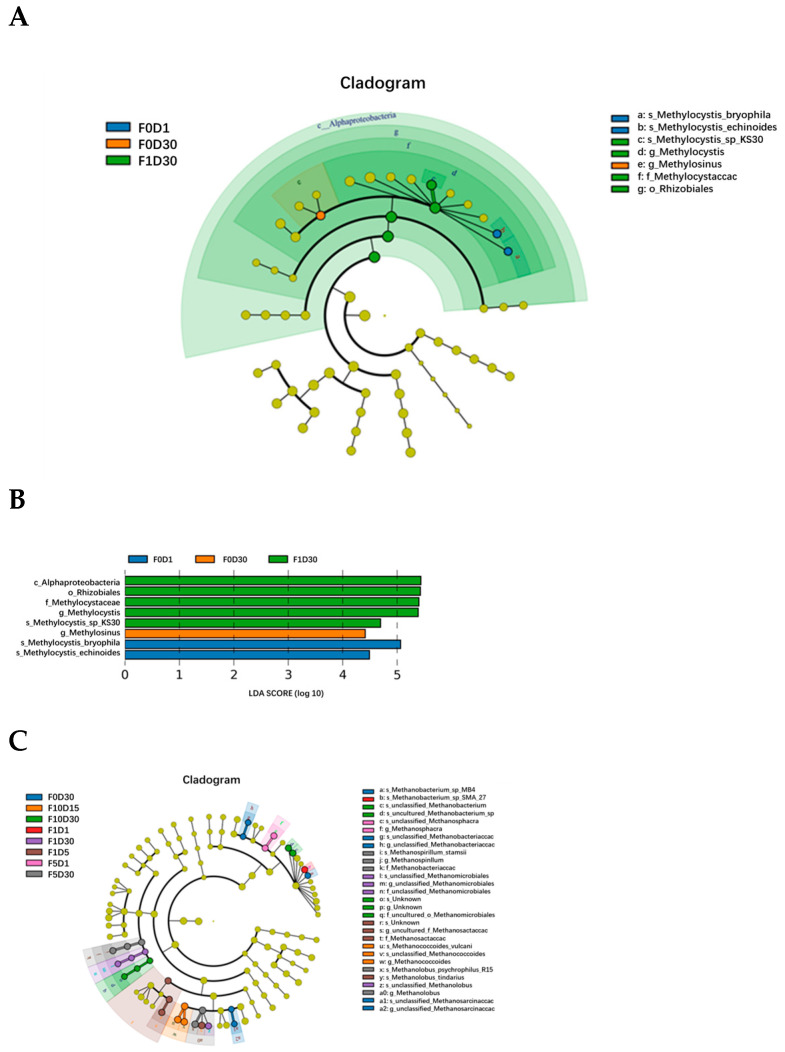

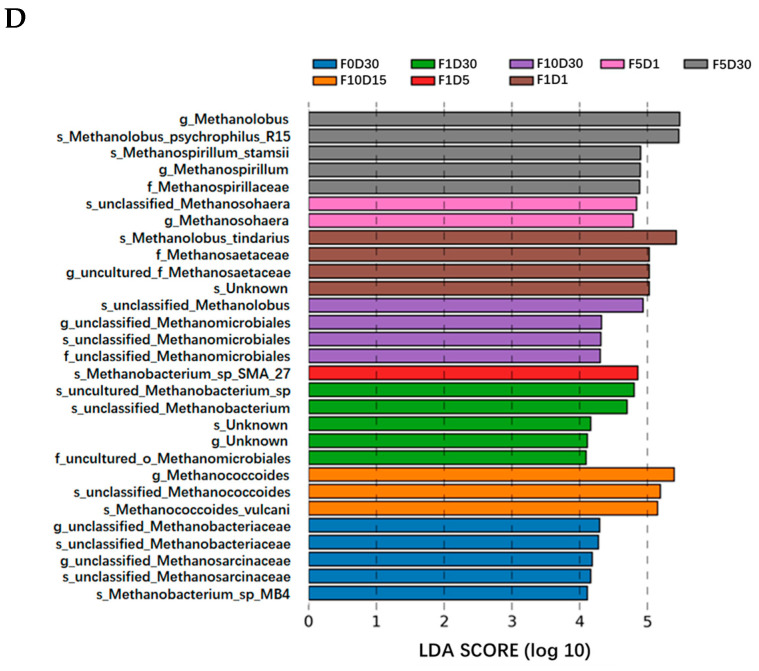
Analysis of the core archaeal of methanotrophs and methanogens. (**A**,**B**) are methanotrophs; (**C**,**D**) are methanogens (AVE ± SD, *n* = 3). Yellow dots: This taxonomic unit shows no significant abundance differences across all experimental groups.

The LEfSe expression profile analysis of microorganisms harboring carbon cycle-related functional genes is as follows (Figure 3). According to the LEfSe analysis, *Methylocystis* and *Methylosinus* were identified as biomarkers for methane oxidation, while *Methanolobus*, *Methanospirillum*, *Methanospirillaceae*, *Methanosphaera*, *Methanosaetaceae*, and *Methanococcoides* were identified as biomarkers for methanogenesis. The random forest modeling revealed that *Methylocystis*, *Methylosinus*, and *Methylococcus* were more influential in the effect of florpyrauxifen-benzyl on methane oxidation (Figure S7A,B). On the other hand, *Methanolobus*, *Methanococcoides*, *Methanogula*, *Methanomassiliicoccus*, *Methanobacterium*, *Methanosphaerula*, *Methanosarcina*, and *Methanosphaera* were found to be of greater importance in the effect of florpyrauxifen-benzyl on methanogenesis (Figure S7C,D). In conclusion, a combination of genus-level species analysis, LEfSe analysis, and random forest modeling revealed that *Methylocystis* and *Methylosinus* played a key role in methane oxidation, while *Methanolobus*, *Methanosphaera*, and *Methanococcoides* played a key role in methanogenesis (Figure 3).

**Figure 4:**
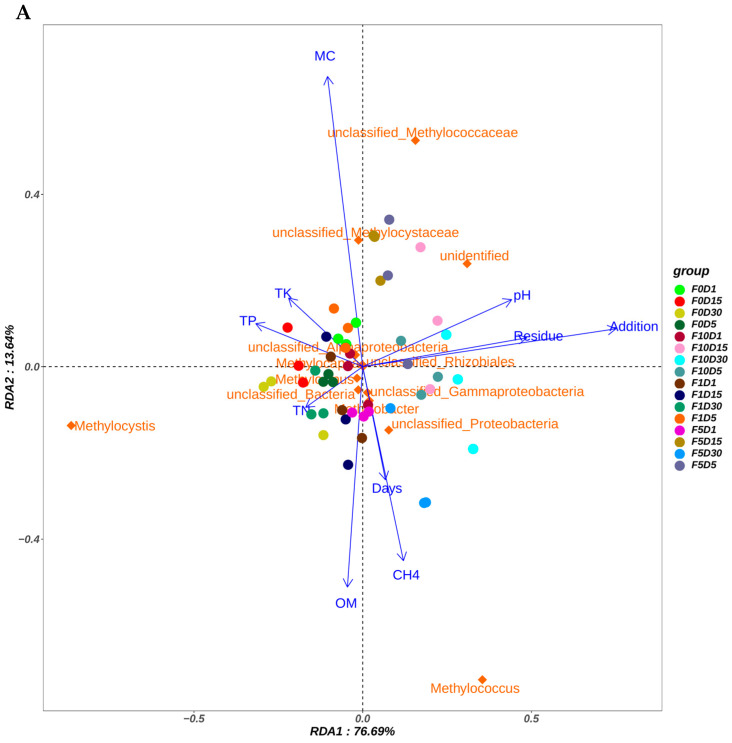

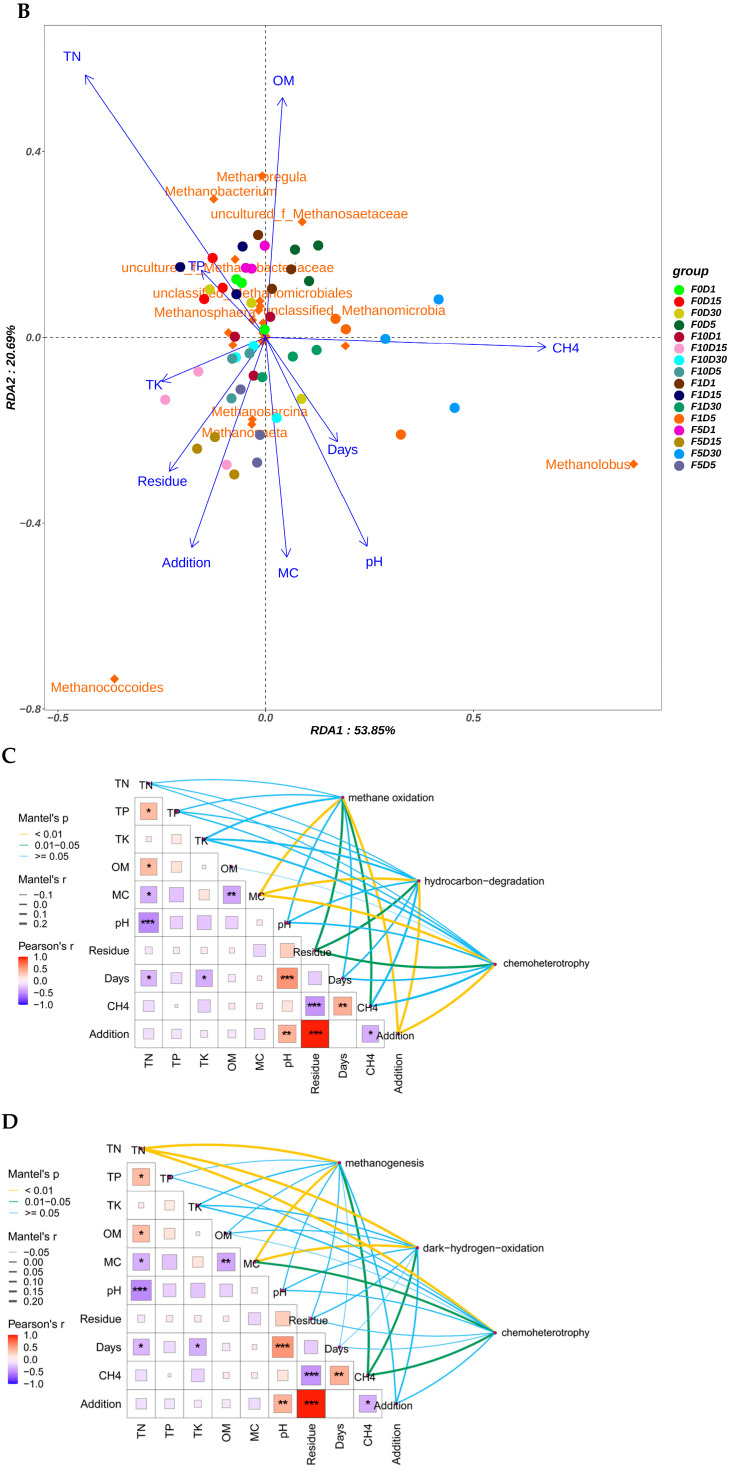
Environmental drivers of methanotrophs and methanogens. (**A**,**B**): redundancy analysis of methanotrophs and methanogens. (**C**,**D**): Spearman’s correlation analysis between environmental factors and microbial community composition of methanotrophs and methanogens. (AVE ± SD, *n* = 3, *** *p* < 0.001, ** 0.001 < *p* < 0.01 and * 0.01 < *p* < 0.05).

The relationships between microorganisms and environmental factors are as follows (Figure 4). Network topology indices showed that interspecific co-occurrence patterns varied considerably between methanotrophs and methanogens (Figures S8 and S9). Methanogens exhibit a more stable network structure compared to methanotrophs, characterized by complex and stable network patterns. Specifically, the network structure of methanogens was found to be tighter under florpyrauxifen-benzyl treatment. Co-occurrence network analysis revealed a greater diversity of methanogens under this treatment and increased synergistic interactions among them.

Functional prediction of methane-metabolizing microorganisms harboring the *pmoA* and *mcrA* genes is as follows. Specifically, functions related to methane metabolism and herbicide degradation, such as methane oxidation, methanogenesis, hydrocarbon degradation, and chemoheterotrophy, were selected for analysis. The main functions of methanotrophs are methane oxidation, hydrocarbon degradation, and chemoheterotrophy. The main functions of methanogens are methanogenesis, dark hydrocarbon degradation, and chemoheterotrophy. The experiment employed Mantel’s test and correlation analysis (Figure 4) to examine the relationships. The results showed that, for the methanotrophs flora, all three functions were significantly correlated (*p* < 0.01) with MC and florpyrauxifen-benzyl addition. Methane oxidation exhibited statistically significant differences (*p* < 0.05) in comparison to florpyrauxifen-benzyl residues and methane emissions. Hydrocarbon degradation, chemoheterotrophy, and florpyrauxifen-benzyl residues also showed statistically significant differences (*p* < 0.05). The application rate and residues of exogenous herbicides directly affect three pathways of methane-oxidizing bacteria: methane oxidation, hydrocarbon degradation, and chemoheterotrophy. In the case of the methanogen flora, all three functions were significantly correlated with soil total nitrogen content (*p* < 0.01). Methanogenesis, dark hydrocarbon degradation, and soil water content were also found to be significantly correlated (*p* < 0.01). Furthermore, Methanogenesis, dark hydrocarbon degradation, and chemoheterotrophy were all statistically different from methane emissions (*p* < 0.05). Chemoheterotrophy exhibited statistically significant differences from MC (*p* < 0.05) (Tables S2 and S3). The addition of exogenous herbicides can directly induce changes in soil pH, which in turn trigger a response in soil total nitrogen content. Through this cascade of physicochemical factors, three key microbial functional pathways—methanogenesis, dark hydrogen oxidation, and chemocheterotrophy—are indirectly regulated.

The redundancy analysis and correlation analysis of herbicide residues and environmental factors are as follows. TN was found to be positively correlated with TP and OM (*p* < 0.05). CH_4_ emissions were significantly and positively correlated with days (*p* < 0.01). The pH was significantly and positively correlated with D = days (*p* < 0.001). TN was negatively correlated with MC, TN with days, TK with Days, and addition with CH_4_ (*p* < 0.05). MC was significantly negatively correlated with OM (*p* < 0.01). Residue was significantly negatively correlated with CH_4_ (*p* < 0.001). pH was significantly negatively correlated with TN (*p* < 0.001) (Figure 4; Table S1). Heatmaps of Spearman’s correlation analysis further showed the relationship between methanotrophs and methanogens and environmental variables (Figure S10) and that TN, pH, florpyrauxifen-benzyl residue, and florpyrauxifen-benzyl addition had a greater effect on methane-metabolizing microorganisms. Therefore, MC, TN, pH, florpyrauxifen-benzyl residue, and florpyrauxifen-benzyl addition were the important environmental factors affecting the methanogenic microorganisms in this experiment. This experiment demonstrated that florpyrauxifen-benzyl had a profound effect on methane emissions.

**Figure 5:**
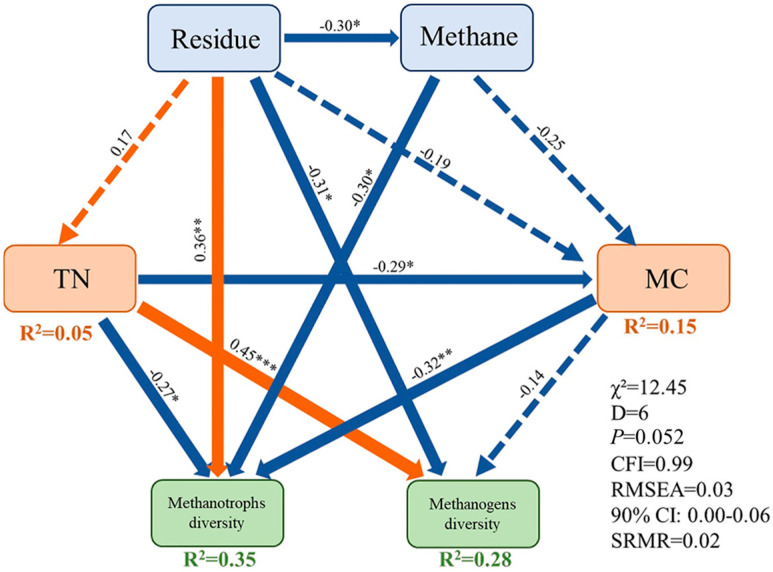
Structural equation models revealing the relationship between treatments, soil physico-chemical properties and CH_4_−metabolizing microorganisms. Orange arrows, positive correlations; blue arrows, negative correlations; bold arrows, strong correlation; thin arrows, weak correlation. Solid lines represent significant (AVE ± SD, *n* = 3, *** *p* < 0.001, ** 0.001 < *p* < 0.01 and * 0.01 < *p* < 0.05) correlations; dashed lines represent non-significant correlations. Coefficients on paths denote standardised path coefficients; R^2^ denotes the proportion of variance explained by each dependent variable.

Structural equation models (SEMs) elucidate the direct and indirect effects of environmental drivers such as herbicide residues on microbial diversity. The results of the structural equations indicated that florpyrauxifen-benzyl was negatively correlated with CH_4_ emissions in the range of 5–10 times the recommended dose. Both florpyrauxifen-benzyl residues and CH_4_ emissions significantly affected the diversity of methanotrophs. A significant negative correlation was observed between florpyrauxifen-benzyl residues and methanogen diversity. In addition, methanotroph diversity was significantly negatively correlated with soil TN. Methanotroph diversity was significantly negatively correlated with soil MC. Methanogen diversity was positively correlated with soil TN, and this relationship was extremely significant. The SEM results confirmed that there was a significant correlation between florpyrauxifen-benzyl residues and CH_4_ emissions (Figure 5).

### 3.4. Effects of Florpyrauxifen-Benzyl on Genes Related to CH_4_ Metabolism

**Figure 6:**
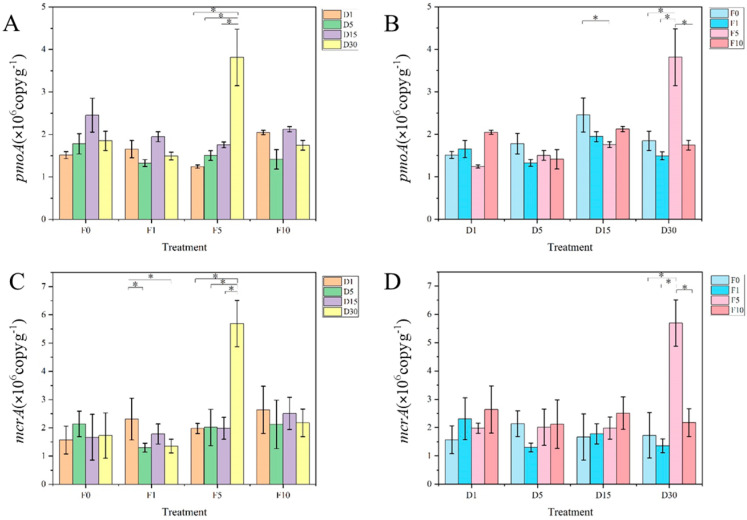
Effect of florpyrauxifen−benzyl concentrations on *pmoA* and *mcrA* gene copy number. (**A**,**B**): copy number of *pmoA* gene on different days under the same concentration of florpyrauxifen−benzyl treatment and the copy number of *pmoA* gene on the same day under different concentrations of florpyrauxifen−benzyl treatments, respectively. (**C**,**D**): copy number of *mcrA* gene on different days under the same concentration of florpyrauxifen−benzyl treatment and the copy number of *mcrA* gene on the same day under different concentrations of florpyrauxifen−benzyl treatments, respectively. (AVE ± SD, *n* = 3, * *p* < 0.05).

Both the *pmoA* gene and the *mcrA* gene have been shown to be associated with CH_4_ metabolism; thus, they can be used as functional genes to assess CH_4_ oxidation and production, respectively. The copy numbers of the *pmoA* and *mcrA* genes at different treatment times and florpyrauxifen-benzyl concentrations are as follows (Figure 6). Over the 30-day experimental period, the copy number of the *pmoA* gene was significantly increased on D30 under the F5 treatment. Among the different dose treatments, the copy number of the *pmoA* gene significantly increased at D30 in the F5 treatment. The *mcrA* gene copy number showed a pattern like that of *pmoA*. The *mcrA* gene copy number on D30 was significantly increased under the F5 treatment. Among treatment doses, the *mcrA* gene copy number significantly increased at D30 in the F5 treatment. Thus, the fluorescence quantitative PCR assay demonstrated that increasing concentrations of florpyrauxifen-benzyl affected the activity of CH_4_-metabolizing microbial communities.

## 4. Discussion

### 4.1. Degradation of Florpyrauxifen-Benzyl in Rice Soils

The study aimed to investigate the elimination of florpyrauxifen-benzyl in rice inter-root soil using ultra-high-performance liquid chromatography (UPLC). Most existing literature reports rely on high-performance liquid chromatography [33]. This experiment will serve as a reference for future research on the degradation of florpyrauxifen-benzyl. Additionally, it will provide a theoretical basis for ecological conservation in cold regions. In aquatic environments, herbicides primarily degrade through hydrolysis and photolysis, while soil microbial degradation is the main mechanism of herbicide dissipation [34,35]. In this test, the degradation of florpyrauxifen-benzyl mainly occurred through microbial degradation. The degradation followed a first-order kinetic equation. The application of florpyrauxifen-benzyl in this experiment caused a sudden change in the soil microorganism environment. Furthermore, the pyridine ring present in florpyrauxifen-benzyl is toxic and inhibitory to microorganisms [36]. Therefore, the addition of florpyrauxifen-benzyl initially inhibited soil microbial activity, but over time, as the microorganisms adapted to the presence of florpyrauxifen-benzyl, they gradually degraded it (Table 1). The functional prediction of methane-metabolizing microorganisms in this experiment also confirmed their ability to degrade florpyrauxifen-benzyl (Figure 4). In a previous study, the dissipation kinetics of florpyrauxifen-benzyl in rice water, rice soil, and rice straw followed first-order kinetics, with half-lives of less than 3 days [37]; however, it yielded different results, possibly because their soil samples were collected from the Agricultural Science and Technology Park at Jiangxi Agricultural University in Nanchang City, Jiangxi Province, China. This region has a warm temperate climate, with an average annual temperature of 17.0–17.7 °C and an annual rainfall of 1600–1700 mm. During our test period, the average temperature was 30.4 °C, with predominantly sunny days, which may have accelerated the dissipation of florpyrauxifen-benzyl [37]. On the other hand, the soil environment in our experiment was in a cold temperate zone, with an average annual temperature of 3.6 °C and an annual rainfall of 569.1 mm. The soil samples used in our experiment were obtained from a colder climatic environment with lower precipitation compared to previous studies. It is well-established that temperature greatly influences the activity of soil microorganisms [38] and increases soil microbial richness [39]. Our experiment utilized cold black soil, which likely harbored different microbial species compared to the previous study. This variation in environmental conditions could have contributed to the differences in half-life and degradation time. Additionally, florpyrauxifen-benzyl may undergo photolysis [40], which could also account for the disparity in degradation times.

### 4.2. Impact of Florpyrauxifen-Benzyl on CH_4_ Emissions

Methane emissions depend on the balance between CH_4_ oxidation and generation. An increase in net CH_4_ emissions was observed in the present study, and the CH_4_ generation pathway produced more CH_4_ than the CH_4_ oxidation pathway (Figure 1). Studies have shown that increased carbon input may make the soil microbial environment favorable for increased CH_4_ emissions [41,42]. This is likely one of the reasons for the increase in net CH_4_ emissions due to the increased application of florpyrauxifen-benzyl. The activity of the CH_4_-metabolizing microorganisms in the soil was affected by the increased application of florpyrauxifen-benzyl, resulting in fluctuations in cumulative and net CH_4_ emissions. During the first 10 days of the experiment, methane emissions followed the order F0 > F1 > F5 > F10. This trend may be attributed to the inhibitory effect of the pyridine ring on microbial activity. It is known that microorganisms adapt to their environment, and once they adapted to an environment containing florpyrauxifen-benzyl, methane emissions started to fluctuate. During days 10–20 of the experiment, both methanogens and methanotrophs in the soil exhibited the ability to degrade florpyrauxifen-benzyl (Figure 4). This phenomenon is hypothesized to result from the ability of soil methanogens and methane-oxidizing bacteria to utilize florpyrauxifen-benzyl as a carbon source for growth, thereby leading to a potential competitive relationship between these two groups of microorganisms. Consequently, methane emissions exhibited a fluctuating state during this period. Additionally, methanotrophs can consume the methane produced by methanogens, which further contributes to fluctuations in the state of methanogens and methanotrophs. At days 20–30 of the experiment, the order F5 > F1 > F10 > F0 was observed, possibly due to the higher content of florpyrauxifen-benzyl in F5 and F10. This high content of florpyrauxifen-benzyl may have inhibited the activity of methanogens and methanotrophs in the early stage. By days 25–30 of the experiment, methanogens and methanotrophs had acclimated to the soil environmental conditions containing high doses of florpyrauxifen-benzyl. Notably, methanogens showed better acclimation to these conditions, resulting in significant methane emissions. This study investigated the activity of methane-metabolizing microorganisms under different conditions (F1, F5, and F10). The results showed that the microorganisms were more active under F5 conditions, which can be attributed to the higher availability of carbon and nitrogen sources compared to F1. Additionally, F5 did not inhibit microbial growth, unlike F10, which had a high concentration of florpyrauxifen-benzyl. It was observed that methane-metabolizing microorganisms developed a “microbial response mechanism” during the application of florpyrauxifen-benzyl [42]. This adaptation could explain the increased methane emissions observed when florpyrauxifen-benzyl was applied at the usual amount. Furthermore, the methane emission under the F5 treatment was found to be significantly higher than in the other treatment groups (Figure 1).

The effects of various herbicides on methane emissions were investigated. Specifically, the application of prosulfuron, a sulfonylurea herbicide, was found to increase N_2_O emissions and CH_4_ consumption, but did not significantly affect CO_2_ emissions. The degradation of the active ingredient of prosulfuron may result in the production of small carbon species, such as acetate, which could explain the enhanced CH_4_ oxidation observed in soils treated with prosulfuron. These findings suggest that the presence of prosulfuron leads to an increase in acetate, which is utilized by methanotrophs for growth [42,43,44]. The increase in nitrifying flora observed in this study may have contributed to the higher rate of CH_4_ consumption. This is because CH_4_ and NH_4_^+^ have similar sizes and shapes, and ammonia monooxygenase does not have a high level of specificity during the initial stages of nitrification. Nitrifying bacteria can also co-oxidize CH_4_. Furthermore, the presence of sulphuryl groups in the structure of prosulfuron, compared to florpyrauxifen-benzyl, may result in the formation of sulphate during the degradation of prosulfuron. Sulphate-reducing bacteria are known to play a specific role in the anaerobic oxidation of methane [45]. The network structure of methanotrophs in the soil environment was found to be less synergistic and simpler compared to that of methanogens. However, when stimulated by florpyrauxifen-benzyl, the network structure of methanogens became more complex and the synergism between species increased. Consequently, methanogens produced more methane, leading to an increase in methane emissions upon florpyrauxifen-benzyl treatment (Figures S7 and S8). Moreover, it was observed that direct interspecies electron transfer is the primary mechanism of methane oxidation, and the application of herbicides results in a decrease in soil redox potential and an increase in methane emissions [44]. It is possible that electron transfer between methanotrophs and methanogens plays a significant role in the observed rise in methane emissions [46].

### 4.3. Effects of Florpyrauxifen-Benzyl on the Structure of Microbial Communities for CH_4_ Metabolism

The experiment aimed to identify methane-metabolizing microorganisms in rice inter-root soil using high-throughput sequencing of carbon cycle functional genes. Methanotrophs and methanogens displayed contrasting trends in the Shannon index after the application of florpyrauxifen-benzyl. The Shannon index of the methanotroph community showed an increasing trend with changes in florpyrauxifen-benzyl dosage, indicating a diversification of microbial communities. Methanogens are strictly anaerobic, whereas methanotrophs (especially aerobic methanotrophs) require oxygen. Enhanced methanogenic activity and increased methane production imply a more stable or expanded anaerobic condition, which compresses the microenvironments where oxygen can diffuse in the soil. This reduces the aerobic niches available to methanotrophs, leading to decreased abundance and diversity of oxygen-sensitive methanotrophic groups [47]. On the other hand, the Shannon index of the methanogen community exhibited a decreasing trend with changes in florpyrauxifen-benzyl dosage. This suggests that populations occupying dominant ecological niches appeared in methanogen colonies, leading to a specific development of the microbial structure [45]. Herbicide application often temporarily inhibits the activity of aerobic heterotrophic bacteria in the soil, and the degradation of certain herbicides can substantially deplete dissolved oxygen in the soil. This localized “hypoxic” environment creates an ideal ecological niche for methanogenic archaea [48]. The ecological responses of methanotrophs and methanogens may differ under the treatment of florpyrauxifen-benzyl (Figure 2). Under florpyrauxifen-benzyl treatment, *Methylocystis* and *Methylococcus* were the dominant methanotrophs, while *Methanoregula*, *Methanobacterium*, *Methanolobus*, *Methanococcoides*, *Methanomassiliicoccus*, *Methanosaeta*, *Methanosarcina*, and *Methanospirillum* were the dominant methanogens (Figure S4). This is consistent with the results of previous studies [46,47,48,49,50]. Florpyrauxifen-benzyl, an aryl pyridine carboxylic acid ester structural herbicide, generates carboxyl groups during degradation. Methanotrophs can utilize these carboxyl groups through the serine cycle to metabolize and oxidize methane [51]. When treated with florpyrauxifen-benzyl, there was a significant decrease in *Methylocystis* and a significant increase in *Methylococcus* (Figure S4). *Methylocystis* is a type II bacterium, while *Methylococcus* is a type I bacterium. Type I bacteria employ the r-strategy to tolerate fluctuating environmental conditions, whereas type II bacteria follow the k-life strategy [52]. The addition of florpyrauxifen-benzyl led to an unstable environment, resulting in a decrease in type II bacteria (k-strategy) and an increase in type I bacteria (r-strategy). The methanogenic metabolic pathways for CO_2_ reduction in methanogens, with and without cytochromes, are similar, with only a few enzymatic reactions differing. The CO_2_ reduction methanogenesis pathway utilizes H_2_ as an electron donor. CO_2_ is then passed through the one-carbon carriers methanofuran (MFR), tetrahydromethanopterin (H4MPT), and Coenzyme M (CoM) and reduced stepwise, ultimately resulting in the formation of CH_4_ [53]. In rice soil, rice roots respire anaerobically in an anaerobic environment, producing significant amounts of CO_2_. Methanogens then reduce the CO_2_ to CH_4_ [20]. Microflora can metabolize complex organic substrates through various intermediates, with butyric and propionic acids being particularly important. These acids can be converted to methanogenic precursors such as acetic acid, CO_2_, and H_2_, which are then utilized by methanogens to synthesize methane [54]. Florpyrauxifen-benzyl ester, as an aryl picolinate structure herbicide, produces a carboxyl group during the degradation process, which can be utilized by methane-oxidizing flora, metabolizing the carboxyl group through the serine cycle and oxidizing methane [51]. This study hypothesizes that methanol and carbon dioxide are generated during the degradation of florpyrauxifen-benzyl. Methanogens utilize methanol and CO_2_ for growth, leading to the formation of CH_4_ [33]. This phenomenon could explain the adaptability of methanotrophs and methanogens in environments where florpyrauxifen-benzyl is applied. It is worth noting that all known pathways of florpyrauxifen-benzyl degradation result in the production of CO_2_ [34]. Additionally, florpyrauxifen-benzyl degradation provides methanogens with a greater availability of substances compared to methanotrophs, which may explain their superior growth. This experiment demonstrates that florpyrauxifen-benzyl affects the composition of methane-metabolizing microbial communities (Figure 2, Figure 3 and Figure S4).

Herbicides, as sources of carbon and energy, can be absorbed and converted into their own supply of nutrients by microorganisms, which can affect the ecosystem [55,56]. Most studies on CH_4_ emissions from rice paddies have focused on the addition of different carbon sources. However, there are relatively few reports on herbicides, which are carbon sources that can be used in large quantities in agricultural practices. Studies have shown that the application of fresh manure may exacerbate CH_4_ production in flooded soils compared to the addition of compost and biochar [57]. The addition of glucose has a significant effect on CH_4_ emissions, with organic acids from glucose oxidation acting as substrates for CH_4_ production; however, glucose itself does not produce CH_4_ [58]. Straw return usually stimulates CH_4_ production [59]. Treatment with rice straw resulted in an increase in total CH_4_ production and in the abundance of methanogens archaea, and the increase in methanogens archaea further stimulated the production of additional CH_4_ from soil organic matter [60]. Herbicides are an integral component of agricultural practices. However, little is known about the relationship between herbicides and CH_4_ emissions, and in-depth research is required. Furthermore, cocultures of iron-reducing bacteria and methanogens can reduce CO_2_ to CH_4_ [61]. *Methanosaeta harundinacea* and *Methanosarcina barkeri* can accept electrons to reduce CO_2_ to CH_4_ via electrical connections with *Geobacter metallireducens* [62,63,64]. These studies have shown that methanogens and methanotrophs do not act singly in the soil environment but rather in conjunction with other microorganisms to influence CH_4_ emissions, in which electron donors may play a key role. Subsequent studies will provide further insights into the synergistic effects of florpyrauxifen-benzyl treatment on microorganisms, leading to elevated CH_4_ emissions. The present experiment was conducted under laboratory environmental conditions, and changes in methanogens and methanotrophs in rice soil with the increased application of florpyrauxifen-benzyl need to be further verified in field experiments.

## 5. Conclusions

Elevated methane emissions were observed as a result of applying florpyrauxifen-benzyl to rice inter-root soils. The rice soil treated with 5 times the recommended dose of florpyrauxifen-benzyl showed a significantly higher methane production compared to the other treatment groups. Compared to the methanotrophs, the network structure of the methanogens was more compact under florpyrauxifen-benzyl treatment. With the decrease in florpyrauxifen-benzyl residue, the diversity of methanotrophs is significantly reduced, while the diversity of methanogens is significantly increased, leading to a significant increase in methane emissions. In addition, with the decrease in florpyrauxifen-benzyl residue, it directly affects or indirectly affects the increase in soil moisture content through a decrease in soil total nitrogen, leading to a significant decrease in the diversity of methanogens and a significant increase in methane emissions. In conclusion, florpyrauxifen-benzyl had a significant impact on the microbial communities involved in soil methane metabolism. This study revealed that during the dissipation of soil residues, the herbicide halauxifen-methyl significantly promoted methane emissions from the rice rhizosphere by altering environmental factors, such as soil nitrogen and moisture in the rhizosphere, and regulating the structure and diversity of the methane-metabolizing microbial community. This finding provides a scientific basis for understanding the indirect impacts of pesticide residues on greenhouse gas metabolism in paddy fields and is of great significance for the accurate assessment of pesticide ecological risks.

## Figures and Tables

**Table 1:** Kinetic parameters for the degradation of florpyrauxifen-benzyl in rice inter-root soils.

Days	F1		F5		F10	
	Residue (mg kg^−1^)	Degradation Rate (%)	Residue (mg kg^−1^)	Degradation Rate (%)	Residue (mg kg^−1^)	Degradation Rate (%)
D1	0.091 ± 0.007 a	9.0	0.493 ± 0.007 a	1.4	0.999 ± 0.001 a	0.1
D5	0.068 ± 0.005 b	32.0	0.326 ± 0.032 b	34.8	0.798 ± 0.102 b	20.2
D15	0.044 ± 0.003 c	56.0	0.238 ± 0.010 c	52.4	0.611 ± 0.079 c	38.9
D30	0.038 ± 0.005 c	62.0	0.162 ± 0.026 d	67.6	0.372 ± 0.021 d	62.8
Equation	Y = 0.0818e^−0.029x^		Y = 0.4393e^−0.035x^		Y = 0.991e^−0.033x^	
R^2^	0.8821		0.8936		0.9842	
*T*_1/2_	23.90		19.80		21.00	

Note: F1 is the recommended dose of florpyrauxifen-benzyl (0.1 mg/L), F5 is five times the recommended dose (0.5 mg/L), and F10 is 10 times the recommended dose (1.0 mg/L). Lowercase letters a–d indicate the significance of each column of numbers, *p* < 0.05.

## Data Availability

The original contributions presented in this study are included in the article/Supplementary Material. Further inquiries can be directed to the corresponding author.

## Supplementary Materials

The following supporting information can be downloaded at: https://www.mdpi.com/article/10.3390/microorganisms14061228/s1, Figure S1: Venn and petal diagrams representing the number and characteristics of Operational Taxonomic Unit(OTUs ) in methanotrophs and methanogens under different treatments.; Figure S2: Rank abundance curves of (A) methanotrophs and (B) methanogens; Figure S3: Effect of florpyrauxifen-benzyl on the diversity of methanogens; Figure S4: Circos plot of species relationships at the level of (A) methanotrophs and (B) methanogens Phylum and Circos plot of genus-level species relationships between (C) methanotrophs and (D) methanogens; Figure S5: Effects of florpyrauxifen-benzyl herbicide on the genus-level population distribution of soil methane-metabolizing microorganisms; Figure S6: The phylogenetic trees of methanogenic and methanotrophic bacteria; Figure S7: Random Forest modeling of methane-metabolizing microorganisms; Figure S8: Co-linear network of methanotrophs and Topological index of methanotrophs; Figure S9: Co-linear network of methanogens and Topological index of methanogens; Figure S10: Spearman’s rank correlation between environmental variables and the number of methanotrophs (A) and methanogens (B) genera; Table S1: Correlation analysis of environmental factors; Table S2: Results of the Mantel test for Methanotrophs; Table S3: Results of the Mantel test for Methanogens.
