# Supplementary material for: Effect of Florpyrauxifen-Benzyl on Methane-Metabolizing Microbial Community in Rice Rhizosphere Soil
Source: Microorganisms. 2026 May 29;14(6):1228. doi: 10.3390/microorganisms14061228 (PMC13302863; doi:10.3390/microorganisms14061228)
Supplement: Supplementary file 1 [file microorganisms-14-01228-s001.zip › microorganisms-4279008-supplementary.pdf]

Supplementary material

A total of 3,838,068 and 3,885,603 methanotrophs and methanogens sequences, respectively, were obtained by sequencing on the Illumina NovaSeq platform. The reads were clustered at a 97% similarity level to obtain Operational Taxonomic Unit (OTUs). Methanotrophs and methanogens showed 364 and 466 OTUs, respectively (Fig. S1).

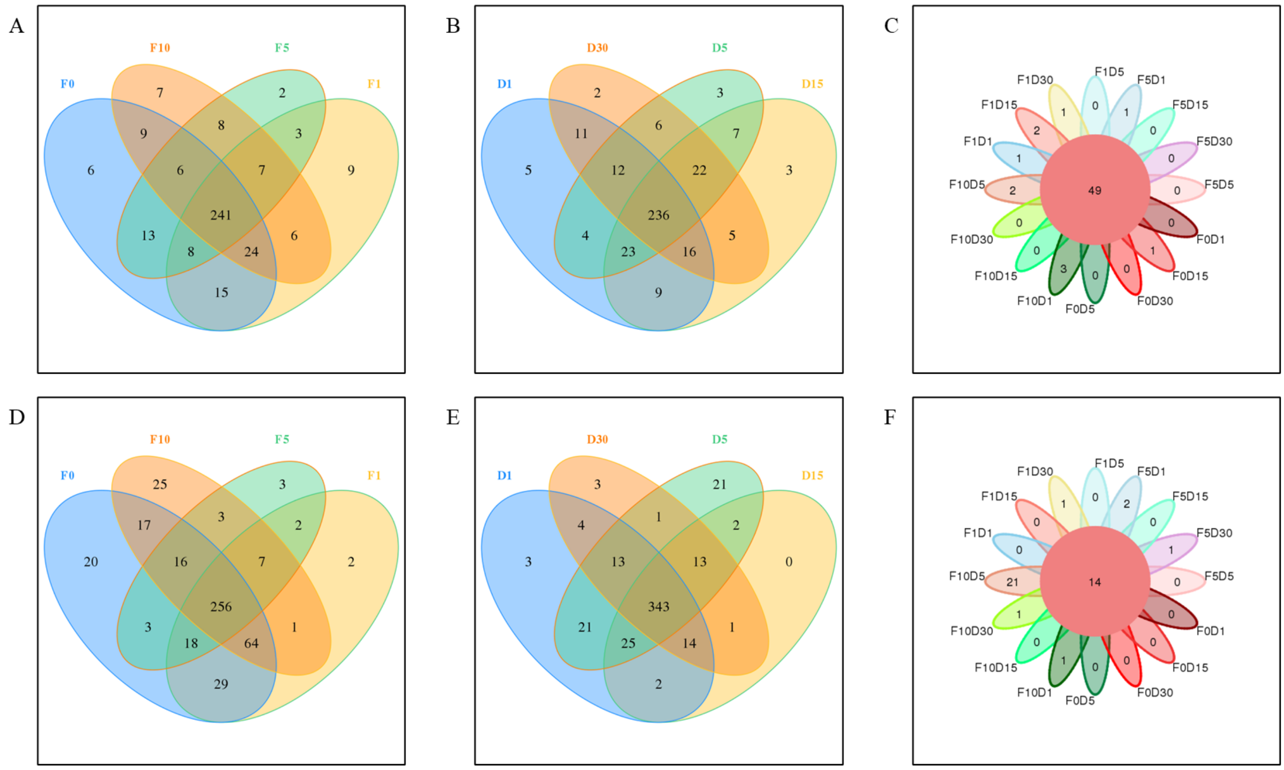

Figure S1 Venn and petal diagrams representing the number and characteristics of Operational Taxonomic Unit(OTUs)in methanotrophs and methanogens under different treatments. A and B: the number of OTUs in methanotrophs under different concentrations of florpyrauxifen-benzyl application and on different treatment days, respectively. D and E: the number of OTUs in methanogens under different concentrations of florpyrauxifen-benzyl application and on different treatment days, respectively. C and F: the characteristic statistic of methanotrophs and methanogens, respectively.

By counting the number of sample sequences at each stage of data processing and assessing the quality of the data, rank abundance curves indicated a high degree of species homogeneity, allowing for subsequent data analyses.

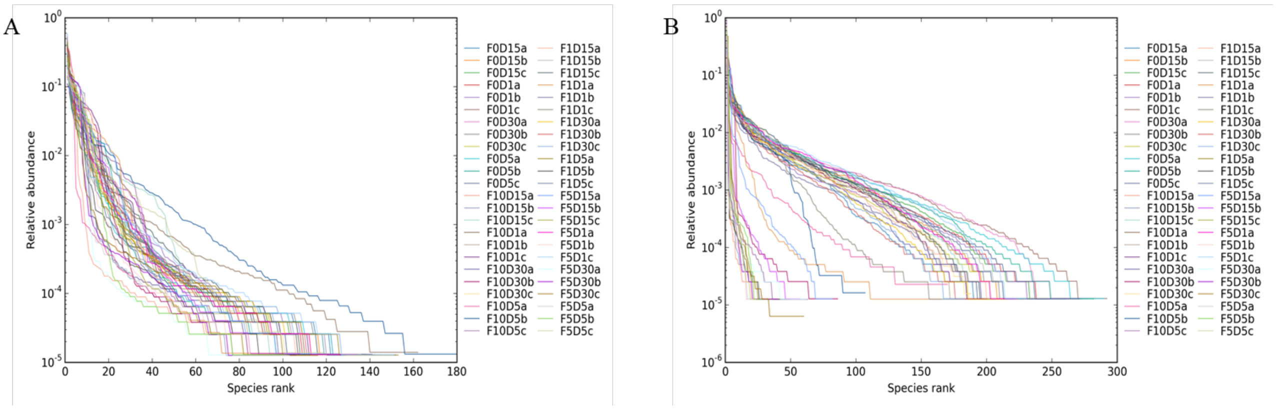

Figure S2 Rank abundance curves of (A) methanotrophs and (B) methanogens.

Moreover, within the same florpyrauxifen-benzyl dose treatment, there was a significant difference between groups for D30 compared to D1 under F5 treatment. Similarly, under F10 treatment, the difference between groups was significant for D15 compared to D1. The Alpha diversity was found to be significantly greater in F10 than in F5, except at D30, where it decreased under the other treatments (Fig. S3).

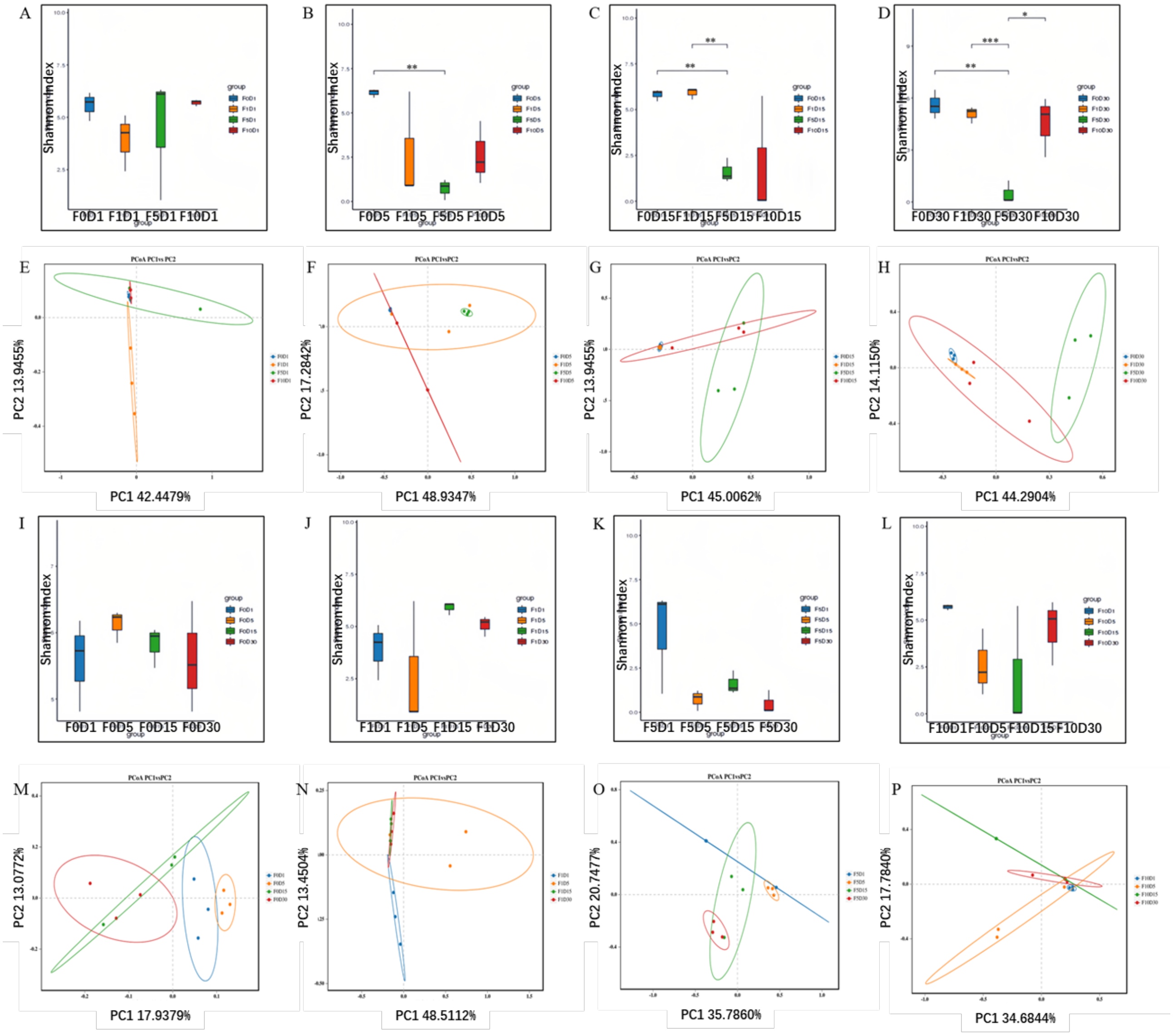

Figure S3 Effect of florpyrauxifen-benzyl on the diversity of methanogens. A, B, C, and D: the effects of increasing concentrations of florpyrauxifen-benzyl on the alpha diversity of methanogens. E, F, G, and H: the effects of increasing concentrations of florpyrauxifen-benzyl on the beta diversity of methanogens. I, G, K, and L: the effect of variation in days on the alpha diversity of methanogens. M, N, O, and P: the effect of variation in days on the beta diversity of methanogens. (**0.001< *p* <0.01 and *0.01< *p* <0.05).

At the Phylum level, the composition of methanotrophs differed significantly from that of methanogens, with the vast majority of methanotrophs in the inter-root soils of the flooded rice fields belonging to Proteobacteria and a very small proportion belonging to candidate-division-NC10 (Fig. S4A). In contrast, all the methanogens in the inter-root soil of the flooded rice fields belonged to Euryarchaeota (Fig. S4B). The dominant genera (relative abundance >1%) among methanotrophs were *Methylocystis* (60.48%) and *Methylococcus* (8.33%) (Fig. S4C). The dominant genera (relative abundance >1%) among methanogens were *Methanoregula* (23.65%), *Methanobacterium* (17.23%), *Methanolobus* (9.90%), *Methanococcoides* (7.38%), *Methanomassiliicoccus* (3.66%), *Methanosaeta* (1.38%), *Methanosarcina* (1.34%), and *Methanospirillum* (1.30%) (Fig. S4D).

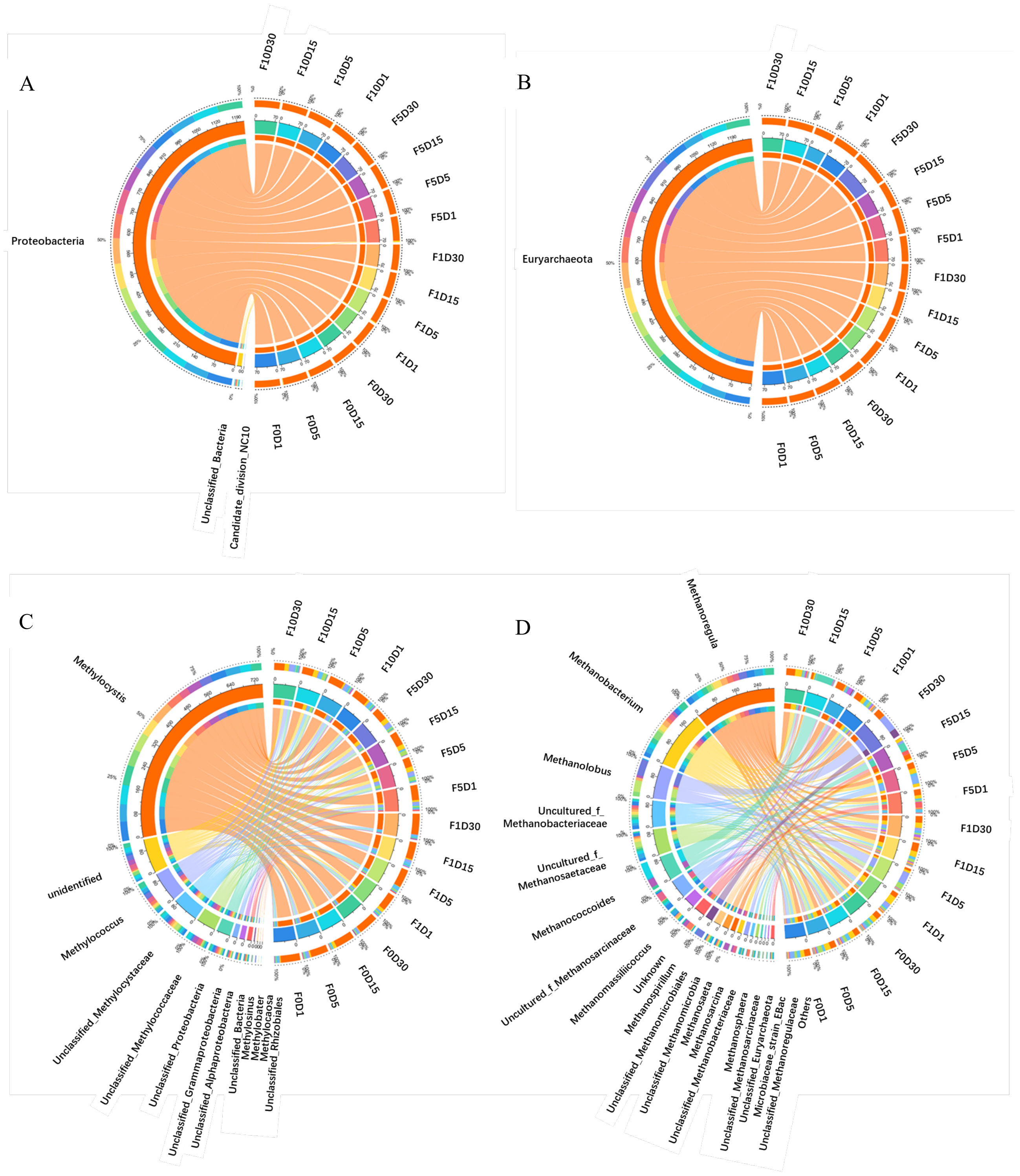

Figure S4 Circos plot of species relationships at the level of (A) methanotrophs and (B) methanogens Phylum and Circos plot of genus-level species relationships between (C) methanotrophs and (D) methanogens.

The experiment analysed the effect of florpyrauxifen-benzyl on the species composition of methane-metabolising microorganisms (Fig. S5). For methanotrophs: A total of five types of methanotrophs were found under herbicide treatment, namely: *Methylocystis*, *Methylococcus*, *Methylosinus*, *Methylobacter*, *Methylocapsa*. The abundance of D15 *Methylococcus* increased significantly under F1 treatment. Among them abundance of D30 *Methylosinus* increased significantly under F5 treatment. At day D15, the number of *Methylococcus* increased significantly and the abundance of *Methylocystis* decreased significantly. At D30 days, the abundance of *Methylocystis* decreased significantly. *Methylocapsa* and *Methylobacter* showed a significant increasing trend in abundance in the F1D5 and F5D30 treatment groups, respectively. (Fig. S5A).

For methanogens: a significant decrease in the abundance of *Methanococcoides* and a significant increase in the abundance of *Methanoculleus*, *Methanolinea*, *Methanogula*, and *Methanosphaera* were observed under F1 treatment. Under F5 treatment, the abundance of *Methanoculleus*, *Methanolinea*, and *Methanomassiliicoccus* decreased significantly, and the abundance of *Methanococcoides* and *Methanolobus* increased significantly. The abundance of *Methanococcoides*, *Methanobacterium* significantly increased under F10 treatment. The number of *Methanococcoides*, *Methanomicrobiaceae_strain_EBac* increased significantly at day D1. At day D5, there was a significant decrease in the abundance of *Methanocella*, *Methanoculleus*, *Methanomicrobiaceae_strain_EBac*, and *Methanosphaerula*. At day D5, the abundance of *Methanolobus* increased significantly. At day D15, there was a significant decrease in the abundance of *Methanoculleus*, *Methanomassiliicoccus*, and *Methanosphaerula*. Significant increase in the abundance of *Methanococcoides*, *Methanogula*, *Methanosphaera*, *Methanospirillum*. At day D30, the abundance of *Methanobacterium* decreased significantly and the abundance of *Methanolobus* increased significantly (Fig. S5B).

A

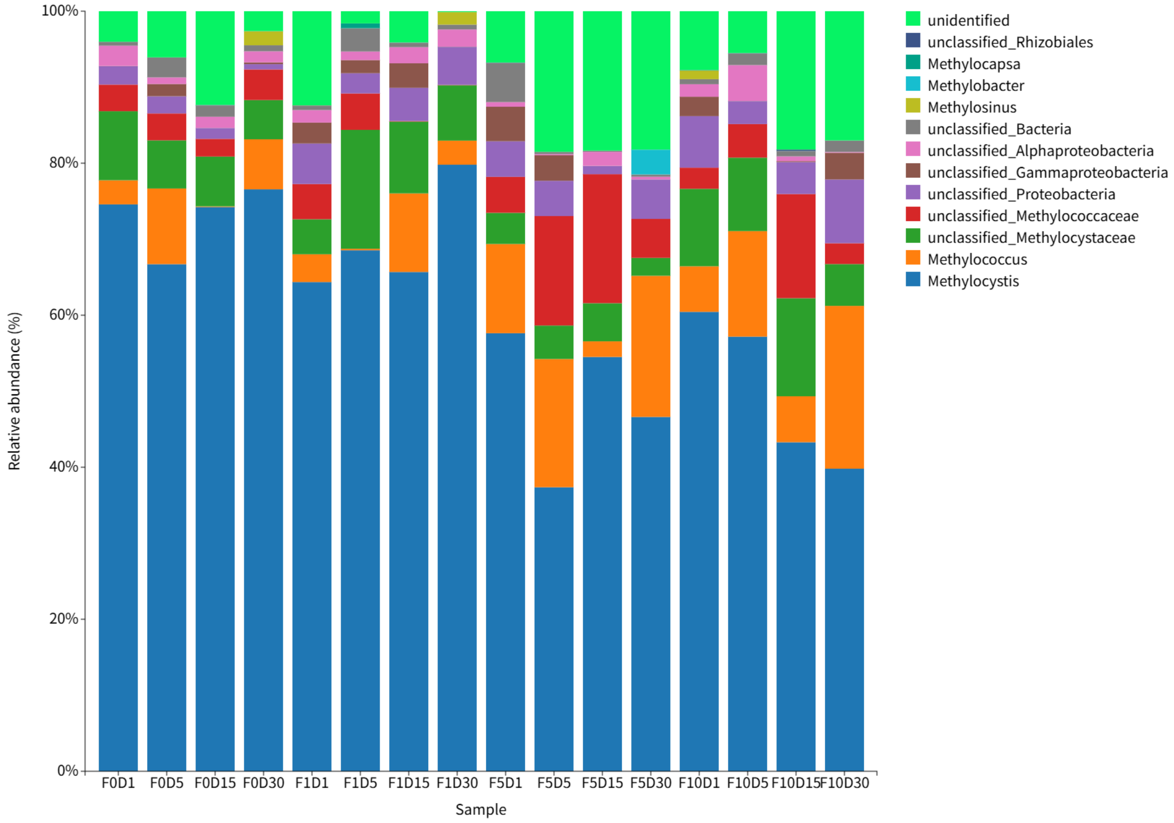

B

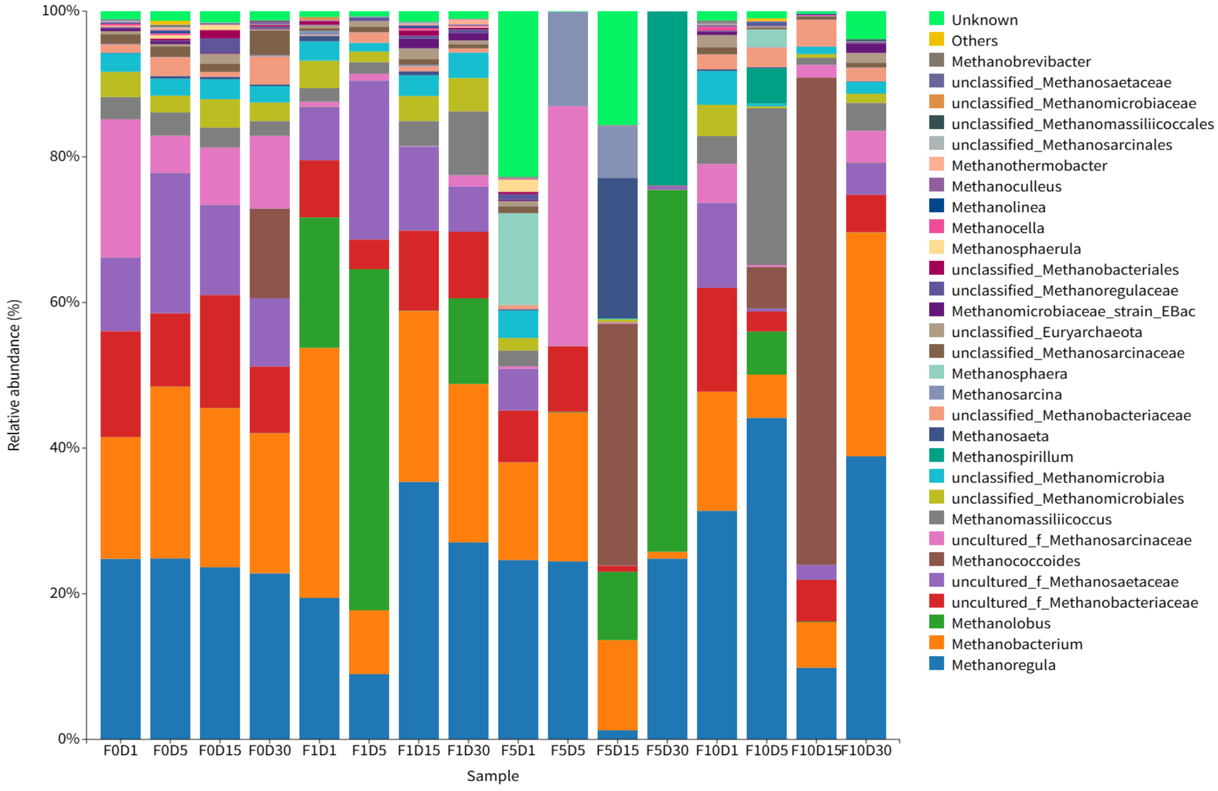

Figure S5 Effects of florpyrauxifen-benzyl herbicide on the genus-level population distribution of soil methane-metabolizing microorganisms. A shows the population distribution of methanotrophs under different application rates of florpyrauxifen-benzyl; B shows the population distribution of methanogens under different application rates of florpyrauxifen-benzyl.

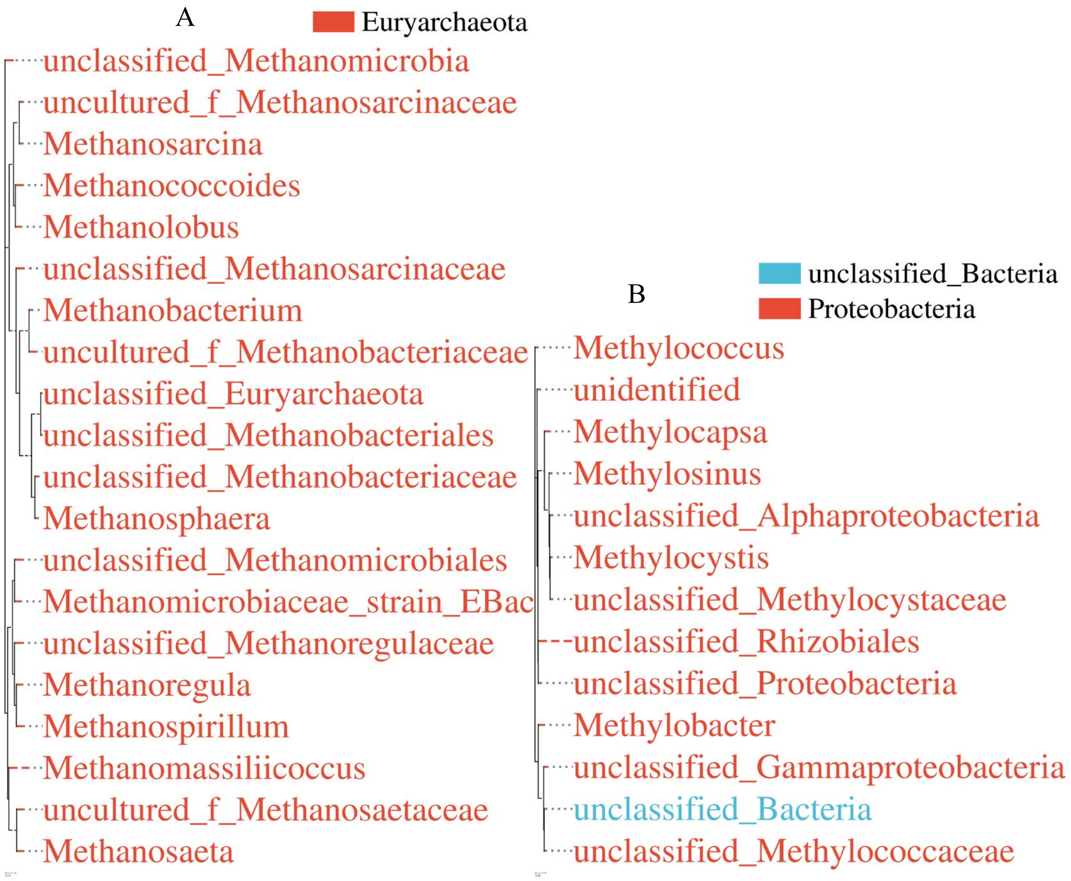

Figure S6 The phylogenetic trees of methanogenic and methanotrophic bacteria.

Note: A: Methanogenic； B: Methanotrophic

According to the LEfSe analysis, *Methylocystis* and *Methylosinus* were identified as biomarkers for methane oxidation, while *Methanolobus*, *Methanospirillum*, *Methanospirillaceae*, *Methanosphaera*, *Methanosaetaceae*, and *Methanococcoides* were identified as biomarkers for methanogenesis. Additionally, a random forest model analysis was performed to study methane-metabolizing microorganisms (Fig. S7). The random forest modeling revealed that *Methylocystis*, *Methylosinus*, and *Methylococcus* were more influential in the effect of florpyrauxifen-benzyl on methane oxidation (Figure S7A, Figure S7B). On the other hand, *Methanolobus*, *Methanococcoides*, *Methanogula*, *Methanomassiliicoccus*, *Methanobacterium*, *Methanosphaerula*, *Methanosarcina*, and *Methanosphaera* were found to be of greater importance in the effect of florpyrauxifen-benzyl on methanogenesis (Fig. S7C, Fig. S7D).

Variance partitioning analysis showed that methanotrophs and methanogens responded differently to environmental factors, with soil physicochemical properties explaining most of the variations in the microbial community. Methanotrophs showed a significant linear correlation with both florpyrauxifen-benzyl concentrations and CH_4 emissions. This confirmed the greater effect of florpyrauxifen-benzyl application multiplicative on the methanotrophs. Network topology indices showed that interspecific co-occurrence patterns varied considerably on methanotrophs and methanogens (Fig. S7; Fig. S8; Fig. S9).

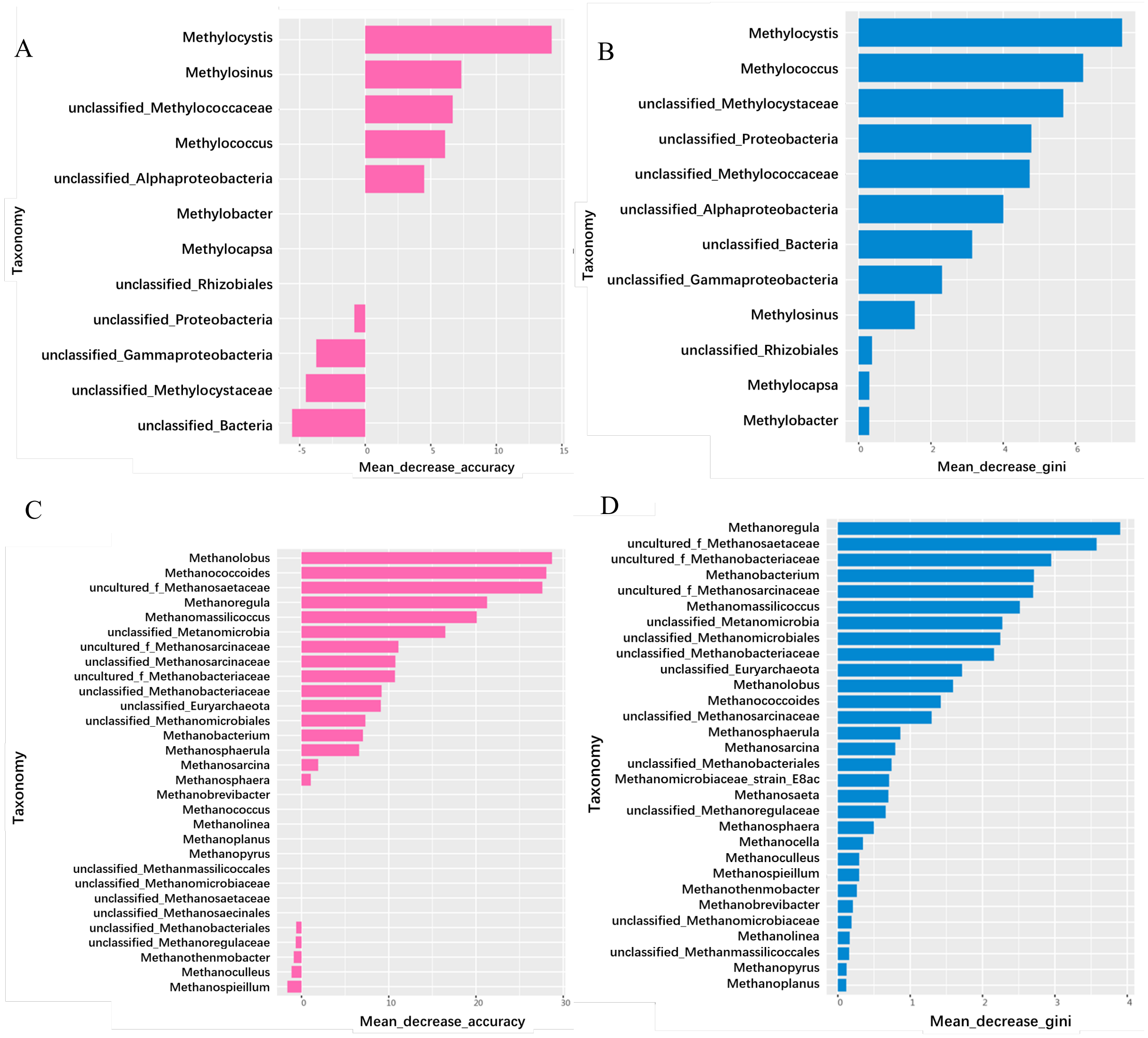

Figure S7 Random Forest modeling of methane-metabolizing microorganisms.

Note: A、B: Methanotrophs and C、D: Methanogens.

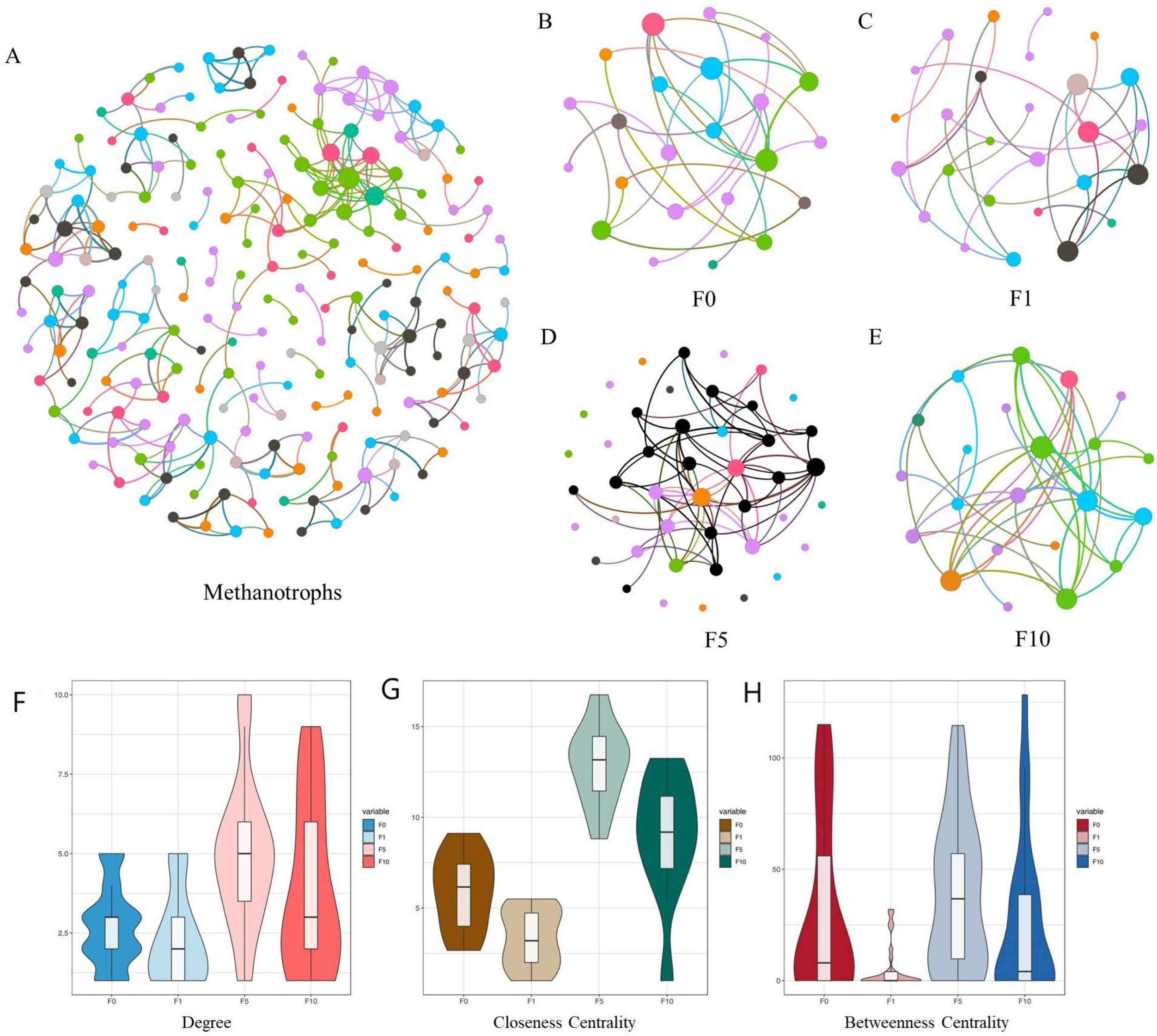

Figure S8 Co-linear network of methanotrophs and Topological index of methanotrophs.

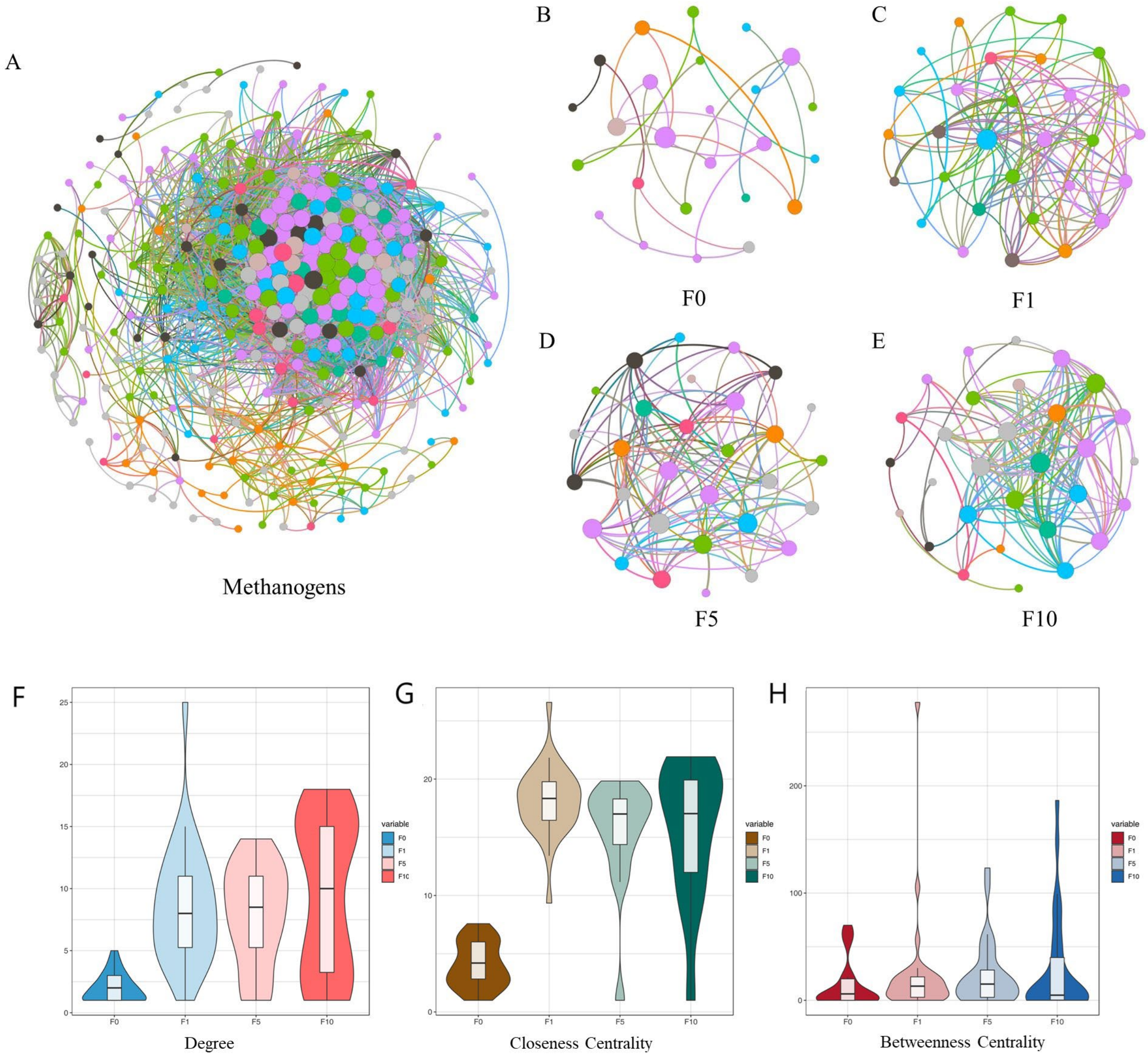

Figure S9 Co-linear network of methanogens and Topological index of methanogens.

Heatmaps of Spearman correlation analysis further showed the relationship between methanotrophs and methanogens with environmental variables (Fig. S10).

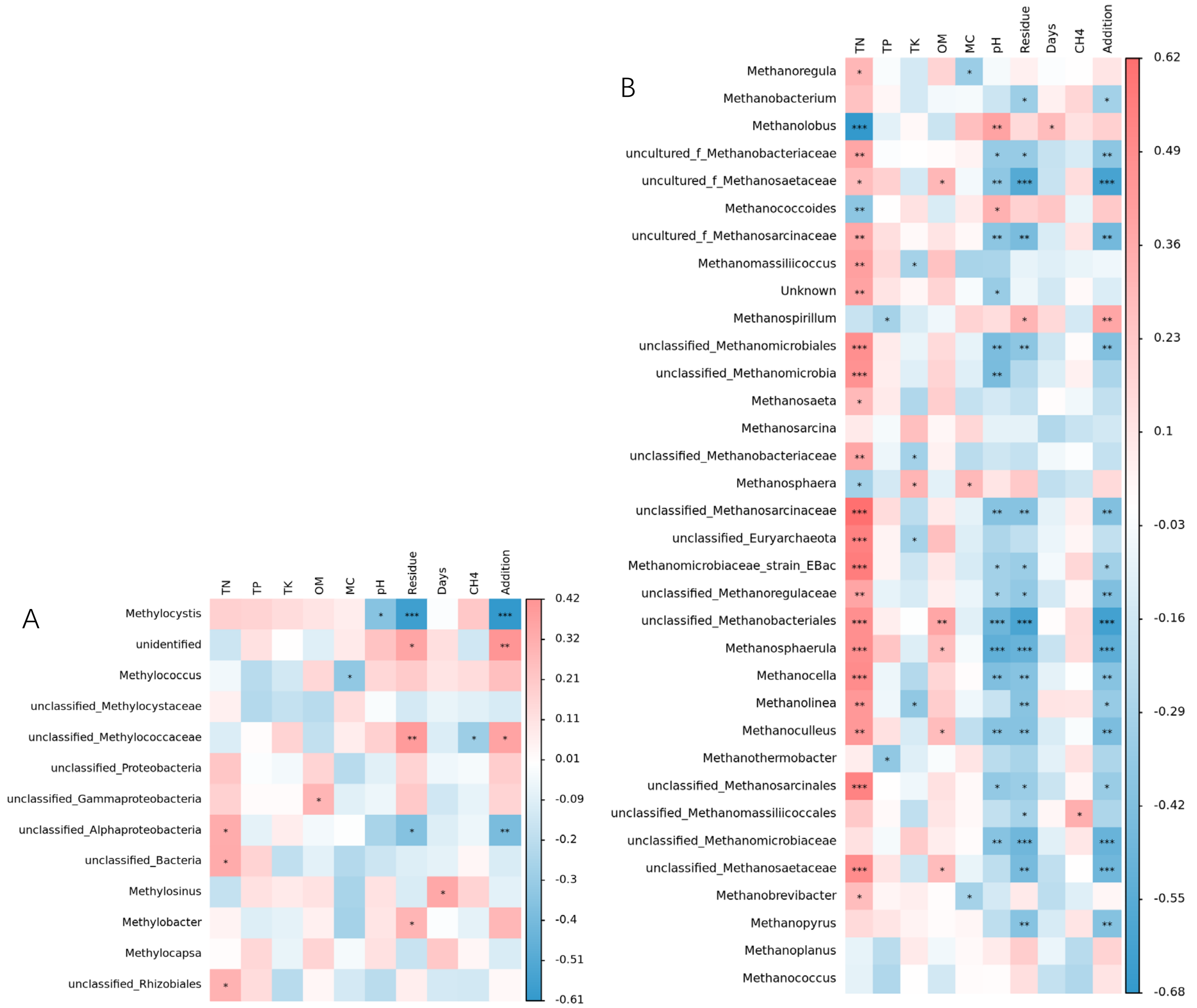

Figure S10 Spearman's rank correlation between environmental variables and the number of methanotrophs(A) and methanogens(B) genera.

Table S1 Correlation analysis of environmental factors.

Correlation	TN	TP	TK	OM	MC	pH	Residue	Days	CH_4	Addition
TN	1									
TP	0.357261	1								
TK	0.035065	0.111597	1							
OM	0.350585	0.144268	0.017858	1						
MC	-0.34717	-0.26541	0.138139	-0.39025	1					
pH	-0.50769	-0.20184	-0.25164	-0.19845	-0.03834	1				
Residue	-0.06343	-0.05131	0.051942	-0.03703	-0.18539	0.257479	1			
Days	-0.31812	-0.15737	-0.35175	-0.06725	0.028247	0.546943	-0.22097	1		
CH_4	-0.15227	0.011784	-0.19881	0.046864	0.041868	0.137267	-0.46329	0.421888	1	
Addition	-0.13787	-0.11702	-0.06255	-0.0807	-0.15468	0.400093	0.962505	0	-0.35931	1

Table S2 Results of the Mantel test for Methanotrophs.

Functions	Environmental factors	R	*p*
Methane oxidation	TN	-0.05007	0.633
Methane oxidation	TP	0.001334	0.462
Methane oxidation	TK	0.132655	0.077
Methane oxidation	OM	-0.13195	0.95
Methane oxidation	MC	0.246603	0.003
Methane oxidation	pH	0.070113	0.2
Methane oxidation	Residue	0.214341	0.018
Methane oxidation	Days	0.072108	0.109
Methane oxidation	CH_4	0.197785	0.047
Methane oxidation	Addition	0.239541	0.001
Hydrocarbon-degradation	TN	-0.05006	0.616
Hydrocarbon-degradation	TP	0.001445	0.458
Hydrocarbon-degradation	TK	0.132485	0.077
Hydrocarbon-degradation	OM	-0.13184	0.949
Hydrocarbon-degradation	MC	0.246665	0.008
Hydrocarbon-degradation	pH	0.070175	0.206
Hydrocarbon-degradation	Residue	0.214279	0.018
Hydrocarbon-degradation	Days	0.072128	0.111
Hydrocarbon-degradation	CH_4	0.197675	0.056
Hydrocarbon-degradation	Addition	0.239349	0.001
Chemoheterotrophy	TN	-0.05028	0.628
Chemoheterotrophy	TP	0.004256	0.448
Chemoheterotrophy	TK	0.124788	0.086
Chemoheterotrophy	OM	-0.11912	0.914
Chemoheterotrophy	MC	0.246653	0.004
Chemoheterotrophy	pH	0.070611	0.207
Chemoheterotrophy	Residue	0.213311	0.018
Chemoheterotrophy	Days	0.068645	0.118
Chemoheterotrophy	CH_4	0.190506	0.062
Chemoheterotrophy	Addition	0.238922	0.001

Table S3 Results of the Mantel test for Methanogens.

Functions	Environmental factors	R	*p*
Methanogenesis	TN	0.239055	0.006
Methanogenesis	TP	-0.01657	0.524
Methanogenesis	TK	0.051912	0.287
Methanogenesis	OM	0.010978	0.451
Methanogenesis	MC	0.195883	0.009
Methanogenesis	pH	0.02308	0.332
Methanogenesis	Residue	0.022864	0.361
Methanogenesis	Days	-0.03227	0.696
Methanogenesis	CH_4	0.210494	0.022
Methanogenesis	Addition	0.07331	0.095
Dark-hydrogen-oxidation	TN	0.244703	0.001
Dark-hydrogen-oxidation	TP	-0.0548	0.705
Dark-hydrogen-oxidation	TK	0.047559	0.291
Dark-hydrogen-oxidation	OM	0.017628	0.416
Dark-hydrogen-oxidation	MC	0.215888	0.005
Dark-hydrogen-oxidation	pH	0.037652	0.315
Dark-hydrogen-oxidation	Residue	0.040492	0.303
Dark-hydrogen-oxidation	Days	-0.03977	0.775
Dark-hydrogen-oxidation	CH_4	0.184268	0.043
Dark-hydrogen-oxidation	Addition	0.086499	0.066
Chemoheterotrophy	TN	0.238271	0.001
Chemoheterotrophy	TP	0.007211	0.429
Chemoheterotrophy	TK	0.061605	0.224
Chemoheterotrophy	OM	-0.03479	0.659
Chemoheterotrophy	MC	0.169055	0.01
Chemoheterotrophy	pH	0.027847	0.344
Chemoheterotrophy	Residue	-0.0059	0.491
Chemoheterotrophy	Days	-0.0172	0.592
Chemoheterotrophy	CH_4	0.188202	0.037
Chemoheterotrophy	Addition	0.041457	0.174
